# Lipidomics Approach Reveals the Effects of Physical Refining Processes on the Characteristic Fatty Acids and Physicochemical Indexes of Safflower Seed Oil and Flaxseed Oil

**DOI:** 10.3390/foods14162845

**Published:** 2025-08-16

**Authors:** Jiayan Yang, Haoan Zhao, Fanhua Wu, Zeyu Wang, Lin Yuan, Yu Qiu, Liang Wang, Min Zhu

**Affiliations:** 1 College of Smart Agriculture (Research Institute), Xinjiang University, Urumqi 830063, China; yangjiayan_m@163.com (J.Y.); yuanlin667788@163.com (L.Y.); 107552303732@stu.xju.edu.cn (Y.Q.); 005448@xju.edu.cn (L.W.); 2 College of Food Science and Technology, Northwest University, Xi’an 710069, China; zhaohaoan@nwu.edu.cn (H.Z.); wufanhua_1@163.com (F.W.); 15032982224@163.com (Z.W.); 3 Bee Product Research Center of Shaanxi Province, Xi’an 710065, China

**Keywords:** safflower seed oil, flaxseed oil, refining, fatty acid, lipidomics

## Abstract

As the principal dietary source of lipids, edible oils (notably vegetable oils) exist in crude form predominantly as triacylglycerols (about 95%), with the remainder comprising impurities and diverse minor components. Therefore, the refining processes of vegetable oil are particularly important. The application potential of safflower seed oil (SSO) in both nutraceutical and pharmaceutical domains is attributed to its exceptionally high linoleic acid concentration and abundant polyphenolic constituents. However, a systematic analysis of SSO during physical refining has yet to be conducted. This study aims to investigate the effects of refining processes on the fatty acids of SSO compared with flaxseed oil (FSO). In this study, chemical analysis, gas chromatography and ultra-high-performance liquid chromatography were used to analyze and compare the physicochemical indexes, fatty acid composition, and the lipidomics of SSO and FSO. Results indicated that optimized refining significantly enhances quality parameters in both SSO and FSO. A total of 40 and 43 fatty acids were identified in SSO and FSO, respectively. Deacidification significantly altered their fatty acid profiles, particularly polyunsaturated fatty acids, with C18:2 and C18:3 being the most affected. A total of 20 significantly different lipids were screened (variable importance in projection > 1.5, *p* < 0.05) and were mainly classified as glycerophospholipids and glycerolipids, of which two lipids (C18:2 and C18:3 (9, 12, 15)) demonstrated particularly marked differences, suggesting that these lipid species represent significant discriminators between SSO and FSO groups; these two lipids exhibited significant alterations during the refining processes of SSO and FSO, respectively.

## 1. Introduction

Edible oil processing is the key to ensuring the quality and safety of food. With the increasing consumer concern for health and nutrition, oil processing technologies are evolving to meet the demand for high-quality edible oils [1]. The refining process of oils removes undesirable components while minimizing damage to desirable constituents and loss of oil, constituting a critical stage in oil processing. For example, changes in fatty acid composition and the nutritional value of edible oil during processing significantly affects oil quality. Edible oils and fats represent plant-derived mixtures primarily constituted by glycerol esters of fatty acids [2]. As fundamental dietary components, edible oils provide significant contributions to human nutrition by serving as important sources of essential fatty acids (EFAs), energy, and fat-soluble vitamins (A, D, E, and K), and diverse nonpolar bioactive compounds [3].

Refining has been extensively investigated for kenaf seed oil [4], avocado oil [5], Papaya (*Carica papaya* L.) seed oil [6], soybean oil [7], sunflower oil [8], and tea seed oil [9]. Saeed M. Ghazani et al. established the indispensable role of refining in edible oil processing [10]. Compared with other edible oils, such as soybean oil, fish oil, sunflower oil, and wheat germ oil, there are few studies on Xinjiang specialty edible oils, which are the special resources in Xinjiang, China.

Xinjiang specialty edible oils include safflower seed oil (SSO), flaxseed oil (FSO), walnut oil, and sunflower oil. As one of the most characteristic edible oils in Xinjiang, existing research on FSO has been relatively well explored, typically focusing on processing techniques such as low-temperature pressing and moderate refining to investigate its compositional characteristics and preserve its bioactive components (e.g., α-linolenic acid and lignans) [11]. However, although SSO is characterized by its high polyunsaturated fatty acid content (predominantly linoleic acid), it lowers low-density lipoprotein (LDL) cholesterol concentrations and reduces atherosclerosis risk, thereby conferring cardiovascular protective benefits; in addition, its α-tocopherol component provides significant antioxidant activity. All of these are widely recognized as critical factors of oil, and SSO remains substantially underutilized [12]. Our team has identified that SSO also holds significant potential as an edible oil, thereby necessitating urgent development and utilization.

Currently, the primary analytical methods for edible oils include chemical analysis, gas chromatography (GC), and liquid chromatography–tandem mass spectrometry (LC-MS/MS), which are utilized to assess the quality of oil. Chemical analysis is employed to determine physicochemical properties such as acid value, peroxide value, and total phenolic content, GC is applied to analyze the fatty acid composition, LC-MS/MS was utilized for fully quantitative lipidomic determination [13,14]. Thus, these established methods were used to investigate the effect of refining processes on the physicochemical characteristics and fatty acids of SSO.

Therefore, in this study, physicochemical, GC, and LC-MS/MS analytical methods were applied to explore basic quality indices and fatty acid composition during different physical refining processes of SSO and FSO, and the lipidomics of refined SSO and FSO, as well as the characteristic fatty acids of the oils, were determined by integrating differential fatty acids before and after, using a lipidomic analysis. The aim of this study is to systematically analyze the effects of refining processes on the physicochemical property and fatty acids of SSO, as well as variations in lipid composition and characteristic fatty acids, with a particular focus on identifying characteristic lipid markers and evaluating their relationship to nutritional properties. These findings would allow for the direct application of the results of the process optimization and oil authentication of SSO.

## 2. Materials and Methods

### 2.1. Materials and Chemicals

The crude SSO and FSO used in this study, obtained by cold-pressing, were provided by Oasis Source Agricultural Technology Co., Ltd. (Xinjiang, China) (Table S1). Trichloromethane was purchased from Gaoyu Fine Chemical Co., Ltd. (Tianjin, China). Methyl tert-butyl ether and acetonitrile were sourced from CNW Technologies (CNW Technologies GmbH, Düsseldorf, Germany). All other reagents utilized in this study were of analytical grade and were purchased from Merck (Darmstadt, Germany).

### 2.2. Refining Process of SSO and FSO

#### 2.2.1. Decolorization

This process was carried out according to the method of Rincón et al. [15], with slight modifications. Crude oil was heated to 65 °C. Softened water (95 °C, 1% salt) was added volumetrically based on crude oil phospholipid content. The mixture was stirred (25–30 rpm, 80 °C) for 40–50 min, centrifugal separation was then performed, and the separated degumming oil was transferred to a drying tower (SHG60; Baoma Grease Co., Ltd., Suzhou, China), where it was heated to approximately 150 °C under vacuum conditions. The oil was heated to 115–120 °C, mixed with clay and activated carbon, then decolorized under vacuum (<−0.095 bar) at 115 °C in a decolorization tower (LYS60; Baoma Grease Co., Ltd., Suzhou, China).

#### 2.2.2. Dewaxing

The decolorized oil was transferred into a dewaxing premix where perlite filter aid was incorporated according to wax-containing molecule levels in the decolorized product. Typically, SSO had an addition rate of eight parts per thousand, while FSO had an addition rate of six parts per thousand. The mixture was transferred to the crystallized oil tank (YYYG140; Baoma Grease Co., Ltd., Suzhou, China), where the temperature was initially maintained at 45 °C and was then gradually cooled to 20 °C after 12 h. In the crystal cultivation oil tank, the temperature was further reduced from 20 °C to 8 °C after 12 h of crystal cultivation, and then filtration was performed. Following transfer to the full filter, the cycle was conducted. After 1.5 h, the mirror was observed to check transparency achievement, and sampling analysis was performed.

#### 2.2.3. Deodorization

The vacuum system was activated. When the vacuum degree reached −0.093 bar, the primary and secondary steams were initiated. When the vacuum degree reached about −0.097 bar, the dewaxed oil was placed into the degassing tower (GZK5O; Baoma Grease Co., Ltd., Suzhou, China). The direct heater was engaged once the oil temperature reached 120 °C. The direct steam was configured to be distributed in an umbrella shape and not as a fog.

#### 2.2.4. Deacidification

For steam deacidification, direct steam was introduced while heating the oil from 120 °C to 150 °C and was dispersed in an umbrella shape rather than as a fog. The oil temperature was cycled from 150 °C to 180 °C for 35–45 min. After oil production, the circulating oil was reheated from 180 °C to 200 °C.

### 2.3. Physicochemical Index Analysis

Oil color was measured using a spectrophotometer (DS-700D, CHNSpec Co., Ltd., Hangzhou, China). The CIE Lab color system was used to determine three oil parameters: lightness (L*), redness (a*), and yellowness (b*). The acid value (AV) was determined according to the cold solvent indicator titration method [16]. The indicator titration method of the National Standards of the People’s Republic of China was used to determine the peroxide value (PV) [17]. The total phenolic content (TPC) was determined via spectrophotometric analysis with Folin–Ciocalteu reagent [18].

### 2.4. Measurement of Fatty Acid Composition

The fatty acid composition of SSO and FSO was determined using GC (Shimadzu, Kyoto, Japan), according to the method of Farhoosh et al. (2008) [19]. After each oil sample was methylated with interesterification [20], 1 mL of 2 mol/L potassium hydroxide solution in methanol was added to the fat extract, followed by vortexing for 1 min and incubation for 10–30 min. Subsequently, 1 mL of 2,2,4-Trimethylpentane was added for extraction. After centrifugation, the supernatant was transferred to a gas chromatography (GC) vial for analysis. The fatty acid methyl esters (FAMEs) were identified using the GC equipped with an SH-Rtx-Wax capillary column (Varian, Palo Alto, CA, USA; 30 m × 0.25 mm i.d, 0.2 μm film thickness) and a flame ionization detector (FID). The inlet temperature was 250 °C and the detector temperature was 280 °C. Nitrogen was used as carrier gas with a total flow rate of 12.3 mL/min and column flow rate of 1.85 mL/min. A split injection mode was used with an injection volume of 1 μL.

### 2.5. Determination of Lipidomics

#### 2.5.1. Metabolites Extraction

Metabolite extraction is conducted first to enable comprehensive metabolite profiling and remove matrix interference. A total of 10 μL of the sample was mixed with 490 μL methyl tert-butyl ether (MTBE). After vortexing for 60 s, the mixtures were sonicated for 10 min using an ice-water bath. Subsequently, 30 μL of the mixture was diluted with 170 μL water, followed by addition of 480 μL extract solution (MTBE/MeOH = 5:1, *v*/*v*) containing the internal standard (SPLASH LIPIDOMIX Mass Spec Standard, Avanti Polar Lipids, Alabaster, AL, USA). The samples were vortex 60 s and re-sonicated for 10 min in an ice-water bath, then centrifuged at 3000 rpm for 15 min at 4 °C. The supernatant (250 μL) of was collected into a fresh tube. To the remaining sample, 250 μL of MTBE was added, followed by vortexing, sonication, and centrifugation, and another 250 μL of supernatant was taken out. This extraction procedure was performed twice in total. And the supernatants were combined and dried in a vacuum concentrator at 37 °C. Then, the dried samples were reconstituted in 100 μL of resuspension solvent (Dichloromethane (DCM)/MeOH/H_2_O = 60:30:4.5, *v*/*v*/*v*), vortexed for 30 s and sonicated for 10 min in an ice-water bath. Following centrifugation (12,000 rpm, 15 min, 4 °C), 35 μL of the supernatant was transferred to a fresh vial for subsequent LC-MS analysis.

#### 2.5.2. LC-MS/MS Analysis

LC-MS/MS analysis was performed according to the method of Want, E.J. et al., 2010 [21]. The UHPLC (ACQUITY Premier, Waters, Milford, MA, USA) separation was carried out using a Nexera LC-40 series UHPLC system. The mobile phase A consisted of 40% water and 60% acetonitrile, while the mobile phase B consisted of 10% acetonitrile and 90% isopropanol (both supplemented with 10 mmol/L ammonium formate). The column temperature was maintained at 45 °C, with the auto-sampler at 10 °C, and the injection volume was 2 μL.

Mass spectrometer (MS) (Triple Quad™ 7500, SCIEX, Ijssel, The Netherlands) was applied for assay development. The typical ion source parameters were as follows: ion spray voltage, +3500/−3000 V; curtain gas, 50 psi; 400 °C; ion source gas, 1:50 psi; ion source gas, 2:50 psi.

Prior to MS analysis, the optimal precursor ion (Q1)/product ion (Q3) pair was selected for each target lipid, and its multiple reaction monitoring (MRM) parameters were optimized.

The mechanical concentration (μg/mL) of each lipid was calculated based on the relationship between the peak area of the internal standard (IS) of the same lipid and the actual concentration. The concentration of the target lipid in the sample (μg/mg) is calculated using Equation (1):

(1) Conc[Spl]=(Conc[Std]×Vol[Extractor])/Amount[Spl]

where *Conc*[Spl] represents the analyte concentration in the sample (μg/mg), *Conc*[Std] denotes the post-run concentration of the standard solution (μg/mL), Vol[*Extractor*] represents the volume of extraction solvent (mL), and Amount[Spl] is the mass of the sample (mg).

### 2.6. Data Analysis

All experimental results were performed with at least three replicates. SPSS 26 statistical software was used to conduct one-way ANOVA, with *p* < 0.05 considered a statistically significant difference. All lipidomic analysis and quantification work in this project was performed using BioBud (v2.0.3). Principal component analysis (PCA) and orthogonal partial least squares-discriminant analysis (OPLS-DA) were carried out in Simca 14.1, with differential lipids identified based on a variable importance in projection value (VIP) > 1.50.

## 3. Results and Discussion

### 3.1. Assay for Effects of Refining Process on Physicochemical Index

#### 3.1.1. Color

**Figure 1:**
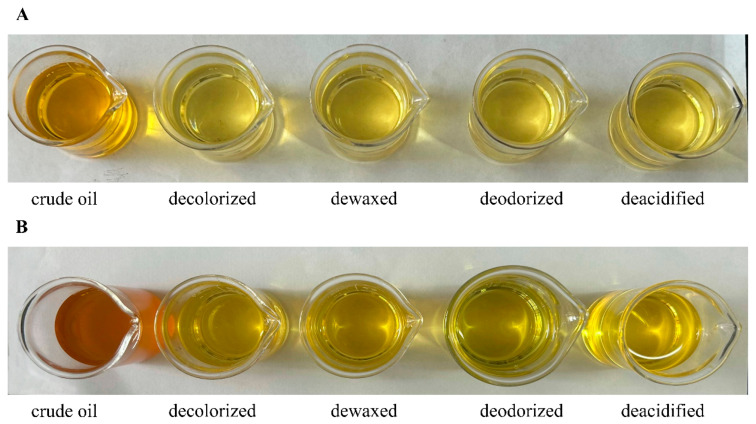
The effect of refining processes on oil color: (**A**) safflower seed oil; (**B**) flaxseed oil.

Color was identified as the predominant sensory factor affecting consumers’ purchasing decisions [22]. The results of color analysis of SSO and FSO were shown in Table S2. Chromaticity differential analysis demonstrated that crude oil exhibited the highest lightness, with no significant variations observed among refined oils. Figure 1 demonstrates that decolorization induced the most significant chromatic alteration in SSO, followed by dewaxing and deacidification. These refining processes resulted in a gradual transformation from brownish-yellow to light yellow, a trend analogous to the chromatic evolution observed in FSO. The observed alterations in in the color of the refined oil may be related to the removal of impurities and the oxidation of oil components during the decolorization process, which consequently leads to a reduction in non-volatile decomposition compounds such as oxidized triacylglycerols and free fatty acids [23].

#### 3.1.2. Acid Value

**Figure 2:**
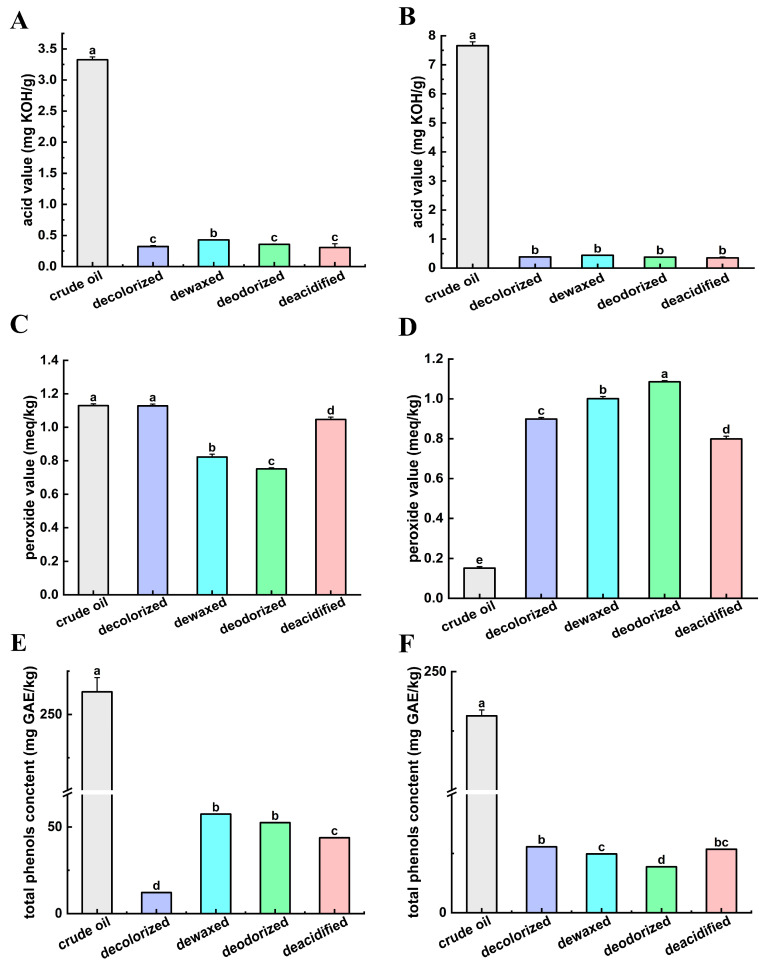
The effect of refining processes on physicochemical index. (**A**) Acid value of SSO. (**B**) Acid value of FSO. (**C**) Peroxide value of SSO. (**D**) Peroxide value of FSO. (**E**) Total phenols content of SSO. (**F**) Total phenols content of SSO. Different letters indicate significant differences among groups.

Acid value (AV) serves as a critical quality parameter of oils, where elevated levels indicate inferior oil quality and advanced rancidity. The AV of SSO throughout different refining processes are presented in Figure 2A, ranging from 3.30 to 0.23 mg KOH/g oil. The crude SSO exhibited the highest AV (3.30 mg KOH/g oil). Similarly, the AV of FSO during refining is shown in Figure 2B, ranging from 7.78 to 0.33 mg KOH/g oil. Both oils exhibited parallel trends in AV reduction during refining, with their respective crude oils showing the maximum values (3.30 mg KOH/g oil for SSO and 7 mg KOH/g oil for FSO). The final refined oils for both SSO and FSO are below the maximum acid value limit specified for vegetable oils in Chinese national food safety standards (≤3 mg KOH/g oil) and the Codex Alimentarius standard (≤0.6 mg KOH/g oil). Specifically, the AV of refined SSO is 0.23 mg KOH/g oil and that of refined FSO is 0.33 mg KOH/g oil [24]. The highest AV was observed in the crude oil. The dewaxed oils showed higher acid values, 0.43 mg KOH/g oil, and 0.44 mg KOH/g oil, in SSO and FSO, respectively. This phenomenon may be due to the fact that the oils were stored for a long time before extraction and a series of oxidative hydrolysis reactions occurred, resulting in higher fatty acids and peroxides in the oils. The decolorization effectively reduced the AV due to the adsorption and removal of impurities by adsorbents. However, the dewaxing process may have released FAs, resulting in a slight increase in the acid value [25].

#### 3.1.3. Peroxide Value

Peroxide value (PV) serves as an indicator for assessing lipid oxidation extent, reflecting oil quality status. Figure 2C depicts the PV of SSO throughout sequential refining process. The PV is characterized by an initial decrease followed by an increase, ranging from 0.7 to 1.1 meq/kg. The PV of the final refined SSO was 0.76 meq/kg. Concurrently, Figure 2D presents the PV of FSO measured after each refining process. A substantial increase in the PV of FSO was observed during the refining process, with values ranging from 0.1 to 1.1 meq/kg. The PV for all samples of both SSO and FSO remained well below the maximum permissible limit (≤10 meq/kg) established by the Codex Alimentarius standards. Specifically, the PV of refined SSO is 1.0 meq/kg and that of refined FSO is 0.8 meq/kg [24]. The PV of the crude oil of SSO was similar to that of decolorized oil but increased significantly after deacidification. It may be due to the deacidification process usually requires the use of lye, and the introduction of lye may cause the oxidation reaction of oil to be aggravated, especially in the presence of high temperature and oxygen. The peroxide value (PV) of FSO was increased during the decolorization process. However, this finding is inconsistent with the vegetable oils. For example, the peroxide value (PV) of both black cumin seed oil and sunflower seed oil (SSO) decreased following the bleaching process. The divergent trends in PV changes may be attributable to differences in the adsorption media (activated carbon and activated clay) and the refining processes (physical refining and chemical refining) [26]. During the decolorization, dewaxing, and deodorization of FSO, elevated processing temperatures combined with higher dosages of clays and activated carbon may accelerate oil oxidation via their extensive surface areas. While effectively removing impurities and deleterious components, this consequently elevates peroxide values (PV) [27].

#### 3.1.4. Total Phenolics Content

Phenolic compounds exhibit significant physiological activities, including cardioprotective, neuroprotective, and antidiabetic effects [28]. Their strong antioxidant capacity is imperative for preserving vegetable oil quality. Polyphenols exhibit potent antioxidant properties mediated through the direct radical scavenging and chelation of metal ions that catalyze free radical formation [29]. Figure 2E shows the total phenolic content (TPC) of SSO during the refining process, ranging from 12 to 265 mg/kg. A significant reduction in TPC was observed throughout the refining process, with the crude oil exhibiting the highest value (265 mg/kg). Similarly, FSO showed a substantial decrease in TPC during refining (Figure 2F), ranging from 19 to 230 mg/kg. The differences in the TPC of the vegetable oils may be attributed to variations in processing and refining conditions. Crude oils exhibit a higher total phenolic content (TPC) than refined oils, which may be due to natural polyphenols’ variability regarding extraction conditions and their significant thermal degradation during high-temperature refining process [30].

### 3.2. Assay for Effects of Refining Process on Fatty Acid Composition

**Figure 3:**
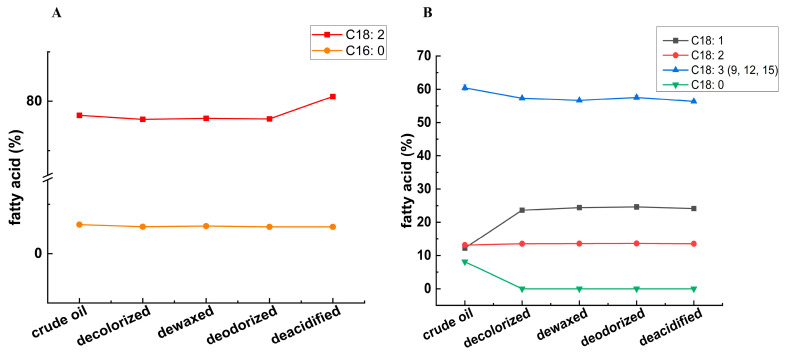
Screening results of significantly different fatty acids (FAs) in oil samples during refining. Differential FAs in SSO (**A**) and FSO (**B**) during refining process. (**A**) Red squares—C18:2. Orange circles—C18:2. (**B**) Blue triangles—C18:3 (9, 12, 15). Red circles—C18:2. Gray squares—C18:1. Green triangles—C18:0.

To explore changes in fatty acids (FAs) of SSO and FSO during refining process, we compared the content of FAs after deacidification and performed multivariate statistical analysis on the extensive dataset (Tables S3 and S4). The threshold VIP > 1 and *p* < 0.05 were used to screen for significant differences in FAs after refining (Tables S5 and S6). Throughout the refining process, SSO exhibited the most significant changes in the levels of fatty acids C18:2 and C16:0, while FSO showed the most significant changes in fatty acids C18:1, C18:2, C18:3 (9, 12, 15), and C18:0. C18:2 displayed significant differences before and after refining in both SSO and FSO (Figure 3). Specifically, its concentration increased significantly during the deacidification of SSO and demonstrated a consistent increasing trend throughout the refining process of FSO. The line graph displays changes in different refining processes. The results demonstrate that the FA content of FSO decreased during the refining process, and the content of C18:3 (9, 12, 15) significantly decreased from 61.45% in crude oil to 56.97% after deacidification. The C18:2 of SSO increased significantly after deacidification, and C18:2 showed an increase from 76.43% to 80.97%. Linolenic acid (C18:3) is widely available in sardine oil [31], Cagaita oil [32], Perilla oil, and FSO [33]. C18:3 is a polyunsaturated ω−3 fatty acid and is required in the human diet as it promotes brain and heart health [34]. Due to the oxidation of multiple double bonds throughout the process, PUFAs, especially linolenic acid, exhibited a decreasing trend [35]. These findings align with classical lipid oxidation theory wherein linoleic and linolenic acids exhibit high autoxidation rates [36]. As an essential fatty acid that cannot be endogenously synthesized in humans, linoleic acid (C18:2) plays a critical role in modulating immune responses, underscoring the significance of its content in edible oils [37].

### 3.3. Lipidomics Analysis of SSO and FSO

Lipids play fundamental roles in biological processes, encompassing diverse physiological functions essential for life activities. Based on established fatty acid differences, we employed lipidomics to comparatively analyze the nutritional lipid profiles of SSO and FSO. To further explore the lipids of SSO and FSO, the multivariate statistical analysis was carried out to obtain and visualize a large set of lipidomics data. A total of 708 lipids were identified in SSO and FSO samples, and these were classified into five lipid categories (fatty acyls [FA], glycerolipids [GL], sterol lipids [ST], sphingolipids [SP], and glycerophospholipids [GP]). The lipids are as follows: 26 bis(monoacylglycero) phosphate (BMP), 12 cholesterol esters (CE), 17 ceramides (CER), 31 diacylglycerols (DAG), 26 digalactosyldiacylglycerol (DGDG), 47 free fatty acids (FFA), 27 hexadecanamide (Hex2Cer), 1 lysophosphatidylcholines (LPC), 2 lysophosphatidylethanolamines (LPE), 1 lyso-phosphatidylserine (LPS), 27 monogalactosyldiacylglycerol (MGDG), 16 phosphatidylcholines (PC), 10 phosphatidylethanolamines (PE), 16 phosphatidylglycerol (PG), 36 phosphatidylinositol (PI), 41 phosphatidylserine (PS), 4 sphingomyelins (SM), and 368 triacylglycerols (TAG).

#### 3.3.1. Principal Component Analysis

**Figure 4:**
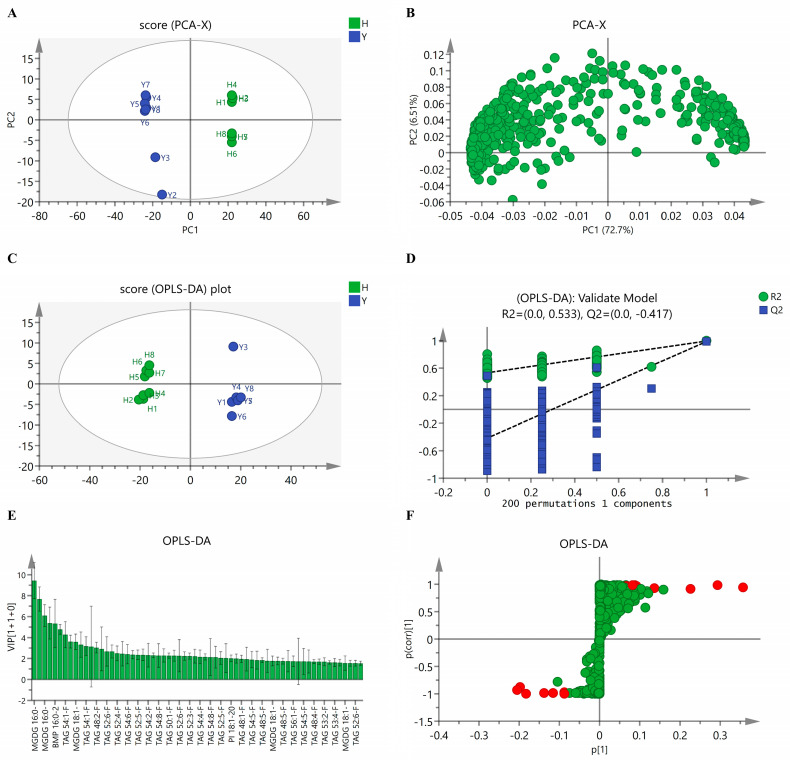
The PCA score plot and loading plot of oil samples. (**A**) Score plot of PCA of SSO and FSO. Blue circles: refined FSO. Green circles: refined SSO. (**B**) Loading scatter plot of oil samples. (**C**) The OPLS-DA score plots of SSO. Blue circles: refined FSO. Green circles: refined SSO. (**D**) Corresponding validation plot between SSO and FSO. Green circles: R2. Blue squares: Q2. (**E**) The VIP of OPLS-DA between SSO and FSO. (**F**) S-plot of OPLS-DA between SSO and FSO. Red circles: notably different lipids.

The principal component analysis (PCA) is a multivariate statistical analysis method that extracts information from original datasets while achieving data simplification through dimensionality reduction. The analyzed data were derived from the peak area of internal standards (IS) and actual concentrations of the same class of lipids in UHPLC. The two initial components (eigenvalue > 1, Q2 > 0.5) accounted for 79.21% of the total variance and were employed to examine the dataset. PC1 represented a contribution ratio of 72.7%, and the other principal component represented 6.51%. As shown in Figure 4A, the PCA of samples corresponded to the refined SSO (the green dots) and the refined FSO (the blue dots). The score plot exhibits differential clustering of oil samples and demonstrates that SSO and FSO were more clustered within the group and that there was a significant difference between SSO and FSO.

As shown in Figure 4B, the loading plot of PC1 and PC2 was utilized to identify dominant discriminating variables between SSO and FSO. PC1 was associated with PI 16:0–24:4 (Phosphatidylinositol), FFA 16:2 (hydnocarpic acid), FFA 14:0 (isomyristic acid), TAG 52:2-FA18:0 (triacylglycerols), TAG 53:2-FA18:2 (triacylglycerols), and TAG 58:6-FA18:0 (triacylglycerols), and the dominant variables of PC2 were PC 16:0–18:3 + AcO (phosphatidylcholines), Cer 18:1–14:0 (C14 ceramide), PI 14:1–22:3 (phosphatidylinositol), TAG 44:1-FA16:1 (triacylglycerols), PE-P 16:0–18:2 (phosphatidylethanolamines), and DAG 14:0–20:0 (diacylglycerols). The contribution rate of the variables is shown in Table S7. As evidenced by the loading plot and contribution ratio, phosphatidylinositol, hydnocarpic acid, and isomyristic acid exerted dominant effects on score distribution.

#### 3.3.2. Discrimination Analysis

The orthogonal projections to latent structures discriminant analysis (OPLS-DA) was used to predict sample categories through 200 permutation tests. The concentrations of lipid components were taken as dependent variables in addition to SSO and FSO samples as independent variables for DA. Figure 4C demonstrates that the SSO samples were located on the left side of the confidence ellipse, distinctly segregated from FSO samples. It is revealed that there are significant differences between SSO and FSO based on the concentrations of lipid components. These differences in lipid profiles may reflect distinct nutritional values, processing stability, and functional health properties between SSO and FSO. The differential lipid signatures serve as biomarkers for investigating species-specific traits across oil sources [38]. The differences in lipids imply variations in the proportion of unsaturated fatty acids, types of tocopherols, and lipid oxidation properties [39]. Analyzing the differences in lipid structure and biological functions can provide a basis for the recombination and processing of lipid nutrients [40]. The permutation testing (*n* = 200) validated the predictability of the OPLS-DA model, indicating that the blue Q2 values to the left (R2 > 0.5) were lower than the original right-positioned points, and the blue regression line of the Q2-points intersected the vertical axis (on the left) below zero (Q2 < 0) (Figure 4D). It is indicated that the model can be effectively used for subsequent analytical applications.

#### 3.3.3. Analysis of the Notably Different Lipids

The VIP and S-plot of the SSO and FSO samples are shown in Figure 4E,F, respectively. The S-plot is a scatter plot of the p [1] versus p (corr) [1] vectors of the predictive component. Its abscissa denotes the loading size of variables on PC1, while the ordinate represents the correlation coefficient (reliability) between variables and PC1. The variable is far from the origin on the “S” curve; that is, the absolute values of p [1] and p (corr) [1] are large, indicating that the variable is the discriminant marker.

#### 3.3.4. Characteristic Different Lipids Compounds in SSO and FSO

**Figure 5:**
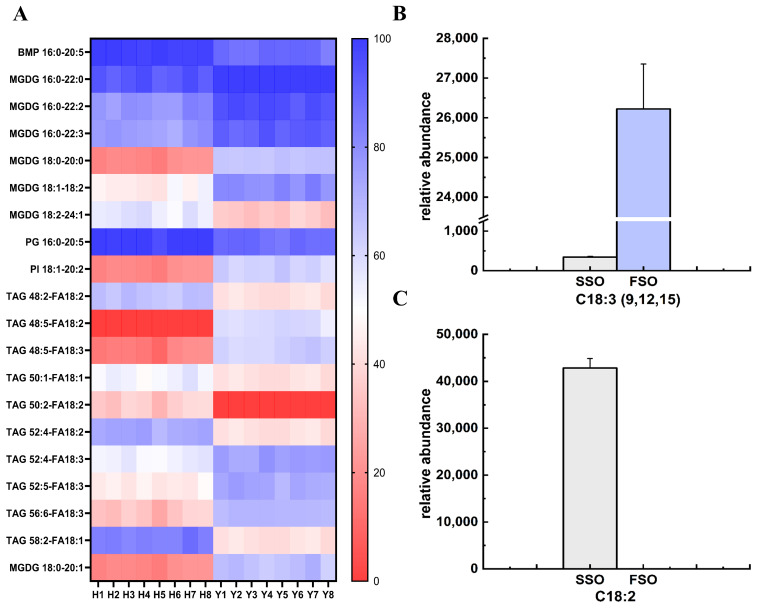
The results of heatmaps of lipids and screenings of FA with significant differences in SSO and FSO. (**A**) Different lipids in SSO and FSO. (**B**) Differential FA expression in SSO and FSO: C18:3 (9, 12, 15). (**C**) Differential FAs expression in SSO and FSO: C18:2.

Multivariate statistics were used to analyze lipidomic data. We combined the two analysis methods (VIP > 1.5, *p* < 0.05) and an S-plot to screen important differential lipid molecules in SSO and FSO as lipid metabolites, and the contents of differential lipids in SSO and FSO are shown in Table 1, while Figure 5A shows the results of screening: a total of 20 significantly different lipids including one BMP molecular species, seven MGDG species, one PG molecular species, one PI molecular species, and ten TAG species. These 20 variables represent significant differences between the SSO and FSO samples analyzed in this study. The heatmap displayed the differing metabolites of the tested oil varieties. The lipid profiles of SSO and FSO were significantly different, and could be used for species identification by lipid molecule species. FSO exhibited significantly lower abundance in most different lipids compared to SSO, except for MGDG 16:0–22:0, MGDG 16:0–22:2, MGDG 16:0–22:3, MGDG 18:0–20:0, MGDG 18:0–20:1, MGDG 18:1–18:2, PI 18:1–20:2, and TAG 48:5-FA18:2, TAG 48:5-FA18:3, TAG 52:4-FA18:3, TAG 52:5-FA18:3, and TAG 56:6-FA18:3. However, several lipid species, including BMP 16:0–20:5, MGDG 16:0–22:0, MGDG 16:0–22:2, and MGDG 16:0–22:3, exhibited relatively higher abundance in both FSO and SSO compared to other lipids. These lipid species not only serve as markers for distinguishing oil types but may also contribute to enhancing immune function, participating in cellular processes, and improving antioxidant capacity in humans. Recent research indicates that BMP plays a key role in the innate immune response in humans, consistent with the high expression levels in macrophages [41]. MGDG exhibits highly efficient and selective anti-inflammatory activity in vivo, with its action depending on the unsaturation of fatty acid chains [42]. PI and its phosphorylated forms are not abundant lipids, but they are important to numerous cellular functions, including cytoskeletal organization, signaling, membrane and lipid movement, and ion channel regulation [43]. PG can act as a bioactive component together with lutein to synergistically protect vision [44]. The structural configuration of TAG governs their oxidative stability, determines the profile of oxidation-derived byproducts and aroma compounds, and fundamentally shapes their physicochemical characteristics [45], which may underpin the differential physicochemical index observed between SSO and FSO in our prior analyses.

Notably, the detection discrepancies of differential lipids and fatty acids between refined SSO and refined FSO exhibited consistency. Employing a metabolomics approach, this study identified lipid differences, specifically in glycerolipids and glycerophospholipids, between SSO and FSO. Gas chromatography verified these differential lipids through comparison with authentic standards, with the simultaneous quantification of their levels. The existence of identical fatty acid species can potentially modulate lipid oxidation and degradation, resulting in differences in lipid biosynthetic pathways [46]. Through comparison with the three differential fatty acids previously identified in unrefined versus refined safflower seed oils, we found that two fatty acids, linolenic acid (LA, C18:2) and α-linolenic acid (ALA, C18:3 (9, 12, 15)), were consistency identified as differential metabolites (*p* < 0.05, VIP > 1) in both analytical groups (Figure 5B,C). The differences in lipidomics lead to variations in FAs, where the triglyceride-bound FAs are LA and ALA. These findings collectively suggest that specific fatty acids, particularly C18:2 and C18:3, show significant differences during the refining process of oils and fats and exhibit content variations in SSO and FSO. Furthermore, our prior findings identified LA and ALA as differential fatty acids that distinguish the refining processes of SSO and FSO. Consequently, we propose that LA and ALA serve as characteristic fatty acids for SSO and FSO.

As an essential polyunsaturated fatty acid (PUFA) in vegetable oils, C18:3 exhibits preventive effects against various metabolic disorders. Importantly, every subclass of ω-3 PUFAs manifests differential and potentially characteristic functionalities in cellular process and metabolic regulation [47]. As an essential ω-6 fatty acid for humans, deficiency in LA may disrupt lipid metabolism, leading to compromised immune function and increased predisposition to cardiovascular disorders [36]. ALA, abundantly present in flaxseed oil, perilla oil, and walnut oil, functions as the primary plant-derived ω-3 PUFA metabolic precursor. ALA can be converted into eicosapentaenoic acid and eicosapentaenoic acid through carbon chain elongation and desaturation, thereby participating in the regulation of cell membrane fluidity, anti-inflammatory responses, and neuroprotection [48]. Previously, olive oil adulteration could be identified by accurately quantifying TAG, and the adulteration of high-priced olive oil and cheap soybean oil, canola oil, or camellia oil could be identified by combining TAG with PCA [49]. As the dominant structural lipid in photosynthetic organisms, MGDG can be efficiently isolated from plants [50]. These differential lipids can be used as a significant difference to distinguish SSO from FSO.

## 4. Conclusions

This study systematically analyzed the effect of the refining process on the physicochemical properties and fatty acids of SSO, as well as its characteristic fatty acids, by examining the physicochemical properties and fatty acids of SSO and FSO during refining. The main results revealed that critical quality parameters of SSO, including acid value and total phenol content, were significantly reduced during the initial refining stage (decolorization), while the peroxide value was not affected by the refining process. The crude oils of SSO and FSO have the darkest colors, and the most significant chromatic alteration was observed after decolorization. Deacidification had the greatest effect on the fatty acid of SSO and FSO, mainly in the polyunsaturated fatty acids, especially the C18:2 and C18:3 isomers. Total quantitative lipidomics analysis of SSO and FSO using HPLC identified 708 lipid species. Multivariate statistical analysis identified 20 significantly different lipids between SSO and FSO, which were primarily classified as bis(monoacylglycero)phosphate, monogalactosyldiacylglycerol, phosphatidylglycerol, phosphatidylinositol, and fatty acids. These differential lipids proved crucial for distinguishing SSO and FSO and could serve as biomarkers to provide a foundation for further research on these single lipids and FSO. These differential lipids could be used to optimize the degree of refining according to the preservation of bioactive components, contributing to the development of functional foods, and providing a theoretical basis for the development of the refining process and the authenticity assessment of SSO. Furthermore, the formation mechanisms of differential lipids should be elucidated in the future.

## Figures and Tables

**Table 1:** The content of differential lipids in SSO and FSO.

Compounds	VIP	S-Plot. p	S-Plot. p (corr)	Content in SSO (μg/mg)	Content in FSO (μg/mg)
BMP 16:0–20:5	5.3393	−0.1986	−0.8806	64.28	35.35
MGDG 16:0–22:0	9.4371	0.3569	0.9500	41.66	129.81
MGDG 16:0–22:2	7.6786	0.2941	0.9786	16.75	74.68
MGDG 16:0–22:3	6.1029	0.2255	0.9114	14.79	51.58
MGDG 18:0–20:0	2.3003	0.0878	0.9830	0.00	5.16
MGDG 18:1–18:2	3.5969	0.1371	0.9356	3.80	16.99
MGDG 18:2–24:1	2.2701	−0.0868	−0.9709	5.76	0.64
PI 18:1–20:2	2.0145	0.0769	0.9601	0.00	4.07
PG 16:0–20:5	5.3846	−0.1986	−0.8806	65.41	35.72
TAG 48:2-FA18:2	3.0391	−0.1164	−0.9882	9.04	0.00
TAG 48:5-FA18:2	1.7521	0.0668	0.9697	0.28	3.33
TAG 48:5-FA18:3	1.8425	0.0696	0.9576	0.85	4.16
TAG 50:1-FA18:1	2.2708	−0.0870	−0.9926	5.02	0.00
TAG 50:2-FA18:2	1.5509	−0.0594	−0.9843	2.40	0.02
TAG 52:4-FA18:2	3.6126	−0.1383	−0.9886	12.75	0.00
TAG 52:4-FA18:3	2.4975	0.0913	0.8663	5.23	11.54
TAG 52:5-FA16:0	2.3542	0.0890	0.9453	2.78	8.32
TAG 52:5-FA18:3	2.3483	0.0899	0.9488	3.51	9.20
TAG 56:6-FA18:3	2.1467	0.0821	0.9808	2.19	6.71
TAG 53:4-FA18:3	1.6159	0.0619	0.9408	1.94	4.64
TAG 58:2-FA18:1	4.7773	−0.1830	−0.9935	22.25	0.00
MGDG 18:0–20:1	2.3674	0.0907	0.9762	0.00	5.58

## Data Availability

The original contributions presented in this study are included in the article/Supplementary Materials. Further inquiries can be directed to the corresponding author.

## Supplementary Materials

The following supporting information can be downloaded at: https://www.mdpi.com/article/10.3390/foods14162845/s1. Figure S1: Schematic diagram of the main process of oil refining; Table S1: The physicochemical index of crude safflower seed oil (SSO) and crude flaxseed oil (FSO); Table S2: The color of SSO and FSO during refining; Table S3: Fatty acids identified in safflower seed oil; Table S4: Fatty acids identified in flaxseed oil; Table S5: Screening of fatty acid differentiators in safflower seed oil; Table S6: Screening of fatty acid differentiators in flaxseed oil; Table S7: Contribution rate of variables on loading plot.
