# Supplementary material for: Lipidomics Approach Reveals the Effects of Physical Refining Processes on the Characteristic Fatty Acids and Physicochemical Indexes of Safflower Seed Oil and Flaxseed Oil
Source: Foods. 2025 Aug 16;14(16):2845. doi: 10.3390/foods14162845 (PMC12386158; doi:10.3390/foods14162845)
Supplement: Supplementary file 1 [file foods-14-02845-s001.zip › foods-3792039-supplementary.pdf]

Supplementary material

Table S1 The physicochemical index of crude safflower seed oil (SSO) and crude flaxseed oil (FSO).

	SSO	FSO
*L**	25.36 ± 0.30	22.59 ± 0.45
*a**	1.92 ± 0.20	0.63 ± 0.14
*b**	4.23 ± 0.41	-0.51 ± 0.37
Acid Value (mg KOH/g)	3.33 ± 0.04	7.6589 ± 0.1334
Peroxide Value (meq/kg)	1.14 ± 0.0052	0.15 ± 0.0037
Total Phenolics Content (mg GAE/kg)	263.68 ± 82.91	231.51 ± 22.82

Table S2 The color of SSO and FSO during refining.

		Crude oil	Decolorized oil	Dewaxed oil	Deodorized oil	Deacidified oil
SSO	*L**	25.36 ± 0.30^{a}	26.68 ± 0.51^{b}	27.29 ± 0.33^{b}	27.33 ± 0.27^{b}	27.57 ± 0.49^{b}
	*a**	1.92 ± 0.20^{a}	0.15 ± 0.04^{b}	0.17 ± 0.02^{b}	0.19 ± 0.02^{b}	0.22 ± 0.05^{b}
	*b**	4.23 ± 0.41^{a}	-1.01 ± 0.27^{b}	-1.03 ± 0.20^{b}	-1.14 ± 0.16^{b}	-1.37 ± 0.11^{b}
FSO	*L**	22.59 ± 0.45^{a}	25.85 ± 1.23^{b}	26.37 ± 0.16^{b}	26.48 ± 0.17^{b}	26.49 ± 0.23^{b}
	*a**	0.63 ± 0.14^{a}	0.08 ± 0.27^{b}	-0.03 ± 0.05^{b}	0.09 ± 0.04^{b}	0.06 ± 0.05^{b}
	*b**	-0.51 ± 0.37^{a}	-0.45 ± 0.18^{b}	-0.63 ± 0.07^{b}	-0.78 ± 0.15^{b}	-0.67 ± 0.24^{b}

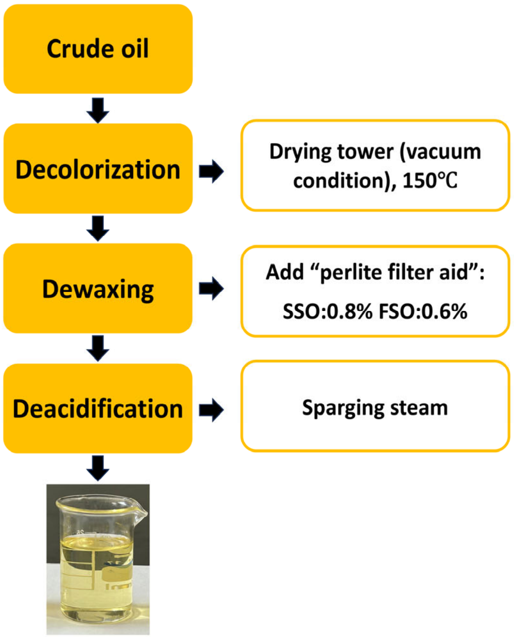

Figure S1 Schematic diagram of the main process of oil refining.

Table S3 Fatty acids identified in safflower seed oil.

**Sample**	**No.**	**Fatty acids**	**Rt (min)**	**Concentration (μg/mL)**
Crude oil	1	C4:0	3.053	3.41
	2	C8:0	5.735	3.34
	3	C10:0	6.582	2.03
	4	C14:0	8.94	30.84
	5	C15:0	9.746	5.07
	6	C15:0	9.838	1.13
	7	C15:0	9.922	1.17
	8	C16:0	10.775	2078.41
	9	C16:0	10.948	7.72
	10	C16:1	11.297	2.56
	11	C17:0	11.774	10.30
	12	C17:0	11.996	6.17
	13	C17:1	12.606	5.69
	14	C18:1	13.538	3540.85
	15	C18:1	13.796	1596.81
	16	C18:3(6.9.12)	14.725	27204.73
	17	C18:3(9.12.15)	15.31	155.19
	18	C18:3(9.12.15)	15.509	1.044
	19	C18:3(9.12.15)	15.816	10.03
	20	C20:0	16.693	135.58
	21	C20:0	16.987	58.422
	22	C20:1	17.563	2.116
	23	C20:2	17.843	12.15
	24	C20:3(8.11.14)	18.733	4.18

	25	C20:3(11.14.17)	19.415	7.76
	26	C20:3(11.14.17)	19.922	18.14
	27	C20:5	20.376	18.18
	28	C22:0	21.327	136.33
	29	C22:0	21.804	65.23
	30	C22:1	22.308	4.38
	31	C22:2	23.131	1.90
	32	C22:2	23.486	10.27
	33	C23:0	24.508	5.73
	34	C23:0	25.107	3.47
	35	C22:6	28.28	62.57
	36	C24:0	28.891	23.96
	37	C24:0	28.929	26.23
	38	C24:1	30.154	6.32
Decolorized oil	1	C4:0	3.05	3.54
	2	C8:0	5.735	2.64
	3	C8:0	5.817	1.27
	4	C14:0	8.939	31.31
	5	C15:0	9.744	6.24
	6	C15:0	9.835	1.19
	7	C15:0	9.92	1.11
	8	C16:0	10.786	2452.40
	9	C16:0	10.952	8.780
	10	C16:1	11.3	2.82
	11	C17:0	11.783	11.73
	12	C17:0	12.001	8.82
	13	C17:1	12.612	6.79
	14	C18:1	13.658	4676.39
	15	C18:1	13.903	2441.32
	16	C18:3(6.9.12)	14.836	34386.4
	17	C18:3(9.12.15)	15.367	199.18
	18	C18:3(9.12.15)	15.564	18.61
	19	C18:3(9.12.15)	15.82	70.48
	20	C20:0	16.741	226.21
	21	C20:0	17.023	75.57
	22	C20:2	17.87	15.75
	23	C20:3(8.11.14)	18.763	5.54
	24	C20:3(11.14.17)	19.445	5.10
	25	C20:3(11.14.17)	19.969	15.44
	26	C20:5	20.413	16.46
	27	C20:5	20.869	5.84
	28	C22:0	21.343	165.79
	29	C22:0	21.819	8.16
	30	C22:1	22.308	2.14

	31	C22:2	23.15	3.06
	32	C22:2	23.518	31.17
	33	C23:0	24.53	7.05
	34	C23:0	25.157	5.58
	35	C22:6	28.283	74.78
	36	C24:0	28.939	71.59
Dewaxed oil	1	C4:0	3.051	3.54
	2	C8:0	5.735	2.86
	3	C8:0	5.813	1.40
	4	C10:0	6.974	2.11
	5	C11:0	7.103	1.80
	6	C14:0	8.939	29.89
	7	C15:0	9.743	6.02
	8	C15:0	9.837	1.17
	9	C15:0	9.918	1.03
	10	C16:0	10.781	2294.54
	11	C16:0	10.948	8.36
	12	C16:1	11.296	3.10
	13	C17:0	11.776	11.22
	14	C17:0	11.997	8.08
	15	C17:1	12.607	6.55
	16	C18:1	13.63	4382.45
	17	C18:1	13.88	2188.04
	18	C18:3(6.9.12)	14.8	32349.93
	19	C18:3(9.12.15)	15.349	191.46
	20	C18:3(9.12.15)	15.549	17.72
	21	C18:3(9.12.15)	15.812	65.45
	22	C20:0	16.727	208.62
	23	C20:0	17.01	69.57
	24	C20:2	17.861	15.48
	25	C20:3(8.11.14)	18.75	5.31
	26	C20:3(11.14.17)	19.439	4.91
	27	C20:3(11.14.17)	19.962	14.60
	28	C20:5	20.404	15.77
	29	C20:5	20.858	5.42
	30	C22:0	21.331	152.89
	31	C22:0	21.818	8.43
	32	C22:1	22.303	2.36
	33	C22:2	23.155	3.24
	34	C22:2	23.512	36.10
	35	C22:2	24	1.67
	36	C23:0	24.519	6.57
	37	C23:0	25.132	4.97
	38	C22:6	28.287	69.21

	39	C24:0	28.917	65.63
	40	C24:1	30.161	3.75
Deodorized oil	1	C4:0	3.051	3.60
	2	C8:0	5.735	3.25
	3	C14:0	8.939	29.70
	4	C15:0	9.744	6.01
	5	C15:0	9.836	1.18
	6	C15:0	9.918	1.10
	7	C16:0	10.781	2313.01
	8	C16:0	10.949	8.27
	9	C16:1	11.297	2.70
	10	C17:0	11.777	11.17
	11	C17:0	11.996	8.30
	12	C17:1	12.609	6.57
	13	C18:1	13.636	4393.80
	14	C18:1	13.855	2305.18
	15	C18:3(6.9.12)	14.807	32680.34
	16	C18:3(9.12.15)	15.351	198.30
	17	C18:3(9.12.15)	15.552	17.76
	18	C18:3(9.12.15)	15.814	66.98
	19	C20:0	16.732	213.46
	20	C20:0	17.012	71.39
	21	C20:2	17.865	15.90
	22	C20:3(8.11.14)	18.753	5.40
	23	C20:3(11.14.17)	19.436	4.97
	24	C20:3(11.14.17)	19.961	14.79
	25	C20:5	20.394	15.74
	26	C20:5	20.864	5.50
	27	C22:0	21.336	156.19
	28	C22:0	21.82	8.00
	29	C22:1	22.31	2.90
	30	C22:2	23.156	3.23
	31	C22:2	23.509	33.88
	32	C22:2	24.005	1.90
	33	C23:0	24.525	6.41
	34	C23:0	25.133	5.33
	35	C22:6	28.306	71.35
	36	C24:0	28.929	67.25
Deacidified oil	1	C4:0	3.048	3.41
	2	C8:0	5.735	3.05
	3	C8:0	5.815	1.14
	4	C14:0	8.938	36.47
	5	C15:0	9.744	7.20
	6	C15:0	9.838	1.51

	7	C15:0	9.917	1.18
	8	C16:0	10.795	2823.39
	9	C16:0	10.955	9.33
	10	C16:1	11.3	3.19
	11	C17:0	11.782	12.86
	12	C17:0	11.997	10.73
	13	C17:1	12.608	7.80
	14	C18:1	13.722	5741.33
	15	C18:2	14.023	2474.04
	16	C18:3(6.9.12)	14.9	39631.70
	17	C18:3(9.12.15)	15.404	269.04
	18	C18:3(9.12.15)	15.591	21.35
	19	C18:3(9.12.15)	15.845	81.34
	20	C20:0	16.754	259.71
	21	C20:0	17.034	86.70
	22	C20:2	17.881	18.94
	23	C20:3(8.11.14)	18.768	6.38
	24	C20:3(11.14.17)	19.454	5.97
	25	C20:3(11.14.17)	19.969	18.48
	26	C20:5	20.411	18.93
	27	C20:5	20.867	6.75
	28	C22:0	21.351	189.38
	29	C22:0	21.836	10.29
	30	C22:1	22.313	3.00
	31	C22:2	23.165	6.12
	32	C22:2	23.519	47.86
	33	C22:2	23.999	2.52
	34	C23:0	24.518	7.88
	35	C23:0	25.138	6.36
	36	C22:6	28.27	84.85
	37	C24:0	28.926	80.86
	38	C24:1	30.151	3.62

Table S4 Fatty acids identified in flaxseed oil.

**Sample**	**No.**	**Fatty acids**	**Rt (min)**	**Concentration (μg/mL)**
Crude oil	1	C4:0	3.063	3.84
	2	C8:0	5.801	11.72
	3	C10:0	6.975	2.42
	4	C11:0	7.091	3.94
	5	C12:0	7.681	1.91
	6	C12:0	7.793	1.15
	7	C14:0	8.939	9.84
	8	C15:0	9.74	5.07
	9	C15:0	9.834	1.51

	10	C15:0	9.917	0.73
	11	C16:0	10.748	1419.43
	12	C16:0	10.933	6.79
	13	C16:1	11.291	1.49
	14	C17:0	11.767	14.81
	15	C17:0	11.99	11.82
	16	C17:1	12.598	3.12
	17	C18:0	13.459	2853.25
	18	C18:1	13.66	4281.18
	19	C18:2	14.346	4636.84
	20	C18:3(9.12.15)	15.658	21636.09
	21	C20:0	16.67	48.62
	22	C20:0	16.97	41.23
	23	C20:2	17.826	8.91
	24	C20:3(8.11.14)	18.709	2.38
	25	C20:4	19.191	11.17
	26	C22:0	21.301	40.21
	27	C22:1	22.466	6.87
	28	C22:2	23.617	18.55
	29	C23:0	24.472	5.99
	30	C22:6	27.602	15.93
	31	C22:6	27.701	9.29
	32	C22:6	27.785	5.97
	33	C22:6	27.898	4.22
	34	C22:6	28.247	80.55
	35	C24:0	28.889	1.28
	36	C24:0	28.989	1.78
Decolorized oil	1	C4:0	3.06	3.79
	2	C8:0	5.80	10.23
	3	C10:0	6.975	3.36
	4	C11:0	7.092	4.21
	5	C11:0	7.211	1.00
	6	C12:0	7.681	1.34
	7	C12:0	7.794	1.43
	8	C12:0	7.909	1.28
	9	C12:0	8.000	1.81
	10	C13:0	8.125	2.65
	11	C13:0	8.204	1.20
	12	C14:0	8.94	11.65
	13	C15:0	9.743	5.86
	14	C15:0	9.835	1.56
	15	C15:0	9.916	0.85
	16	C16:0	10.759	1654.83
	17	C16:0	10.939	22.25

	18	C16:1	11.292	1.94
	19	C17:0	11.77	15.73
	20	C17:0	11.993	13.06
	21	C17:1	12.604	2.87
	22	C18:1	13.5	5004.29
	23	C18:1	13.725	4407.91
	24	C18:2	14.381	5301.43
	25	C18:3(9.12.15)	15.678	22361.20
	26	C20:0	16.454	6.75
	27	C20:0	16.685	77.12
	28	C20:0	16.998	70.60
	29	C20:1	17.369	80.98
	30	C20:2	17.841	10.06
	31	C20:2	18.073	2.70
	32	C20:2	18.353	1.22
	33	C20:3(8.11.14)	18.722	2.57
	34	C20:4	19.201	10.36
	35	C22:0	21.317	46.54
	36	C22:0	21.762	0.92
	37	C22:1	22.148	6.43
	38	C22:2	23.639	12.77
	39	C23:0	24.493	6.27
	40	C22:6	28.249	48.06
	41	C24:0	28.806	11.33
	42	C24:0	29.031	13.44
	43	C24:1	30.101	2.75
Dewaxed oil	1	C4:0	3.056	3.48
	2	C8:0	5.808	7.00
	3	C10:0	6.972	4.42
	4	C11:0	7.093	3.18
	5	C12:0	7.68	1.49
	6	C12:0	7.795	1.12
	7	C12:0	7.907	1.67
	8	C12:0	8.003	1.51
	9	C13:0	8.129	1.11
	10	C13:0	8.204	1.33
	11	C14:0	8.939	12.91
	12	C15:0	9.744	6.46
	13	C15:0	9.835	1.69
	14	C15:0	9.919	0.92
	15	C16:0	10.766	1848.49
	16	C16:0	10.942	25.34
	17	C16:1	11.29	5.19
	18	C17:0	11.773	17.44

	19	C17:0	11.994	14.54
	20	C17:1	12.602	3.27
	21	C18:1	13.533	5852.52
	22	C18:1	13.758	4771.80
	23	C18:2	14.412	5905.37
	24	C18:3(9.12.15)	15.702	24583.42
	25	C20:0	16.462	7.73
	26	C20:0	16.692	80.55
	27	C20:0	17.004	79.03
	28	C20:1	17.378	88.76
	29	C20:2	17.851	10.73
	30	C20:2	18.079	2.87
	31	C20:2	18.346	1.17
	32	C20:3(8.11.14)	18.734	2.76
	33	C20:4	19.21	11.09
	34	C20:3(11.14.17)	19.437	1.62
	35	C20:3(11.14.17)	19.604	1.65
	36	C22:0	21.309	55.40
	37	C22:0	21.798	2.10
	38	C22:2	23.642	13.89
	39	C23:0	24.489	7.36
	40	C22:6	28.256	68.58
	41	C24:0	28.802	15.49
Deodorized oil	1	C4:0	3.055	3.50
	2	C4:0	3.113	2.38
	3	C8:0	5.81	9.26
	4	C10:0	6.975	2.06
	5	C11:0	7.093	2.99
	6	C12:0	7.681	2.32
	7	C14:0	8.939	14.54
	8	C15:0	9.743	7.36
	9	C15:0	9.836	1.91
	10	C15:0	9.918	1.03
	11	C16:0	10.777	2140.41
	12	C16:0	10.947	29.89
	13	C16:1	11.294	1.85
	14	C17:0	11.777	19.66
	15	C17:0	11.994	17.57
	16	C17:1	12.606	3.72
	17	C18:1	13.583	7369.05
	18	C18:1	13.699	1402.41
	19	C18:1	13.824	3558.44
	20	C18:2	14.407	6893.62
	21	C18:3(9.12.15)	15.771	28832.55

	22	C20:0	16.486	7.29
	23	C20:0	16.717	92.02
	24	C20:0	17.03	90.17
	25	C20:1	17.405	103.86
	26	C20:2	17.865	12.46
	27	C20:2	18.098	3.24
	28	C20:2	18.373	1.43
	29	C20:3(8.11.14)	18.745	3.13
	30	C20:4	19.223	12.80
	31	C20:3(11.14.17)	19.465	1.76
	32	C20:3(11.14.17)	19.636	1.76
	33	C20:5	20.39	3.55
	34	C22:0	21.34	63.09
	35	C22:0	21.809	1.72
	36	C22:1	22.439	2.81
	37	C22:2	23.659	15.44
	38	C23:0	24.508	8.44
	39	C22:6	28.281	66.04
	40	C24:0	28.926	16.07
Deacidified oil	1	C4:0	3.056	4.02
	2	C8:0	5.806	7.18
	3	C10:0	6.897	0.97
	4	C10:0	6.974	2.66
	5	C11:0	7.094	3.38
	6	C11:0	7.211	0.85
	7	C12:0	7.68	1.54
	8	C12:0	7.794	1.02
	9	C12:0	7.908	1.13
	10	C12:0	8	1.57
	11	C13:0	8.209	1.65
	12	C14:0	8.94	13.71
	13	C15:0	9.743	6.90
	14	C15:0	9.835	1.82
	15	C15:0	9.918	1.04
	16	C16:0	10.771	1966.72
	17	C16:0	10.944	27.80
	18	C16:1	11.291	3.62
	19	C17:0	11.774	17.99
	20	C17:0	11.994	15.82
	21	C17:1	12.605	3.44
	22	C18:1	13.548	6657.39
	23	C18:1	13.781	4518.70
	24	C18:2	14.437	6267.93
	25	C18:3(9.12.15)	15.736	26304.20

26	C20:0	16.496	7.88
27	C20:0	16.708	83.18
28	C20:0	17.02	82.26
29	C20:1	17.391	94.58
30	C20:2	17.863	11.32
31	C20:2	18.093	3.02
32	C20:2	18.366	1.11
33	C20:3(8.11.14)	18.751	2.56
34	C20:4	19.22	11.73
35	C20:3(11.14.17)	19.465	1.64
36	C20:3(11.14.17)	19.618	1.65
37	C22:0	21.336	55.51
38	C22:0	21.822	2.48
39	C22:2	23.658	15.31
40	C23:0	24.516	6.99
41	C22:6	28.279	58.31
42	C22:6	28.536	13.58
43	C24:0	28.862	18.32
44	C24:1	29.947	2.55

Table S5 Screening of fatty acid differentiators in safflower seed oil.

Compounds	VIP	S-Plot. p(corr)
C18:3(6.9.12)	4.53286	0.970747
C18:2	1.8078	0.998437
C16:0	1.16595	0.955177
C18:3(9.12.15)	0.495088	0.992951
C20:0	0.447805	0.980525
C18:1	0.371098	-0.352042
C22:2	0.249902	0.99911
C24:0	0.22441	0.986486
C22:6	0.172735	0.892788
C22:0	0.148717	-0.708456
C20:5	0.118009	0.988223
C14:0	0.110853	0.955206
C23:0	0.0935131	0.936526
C17:0	0.0908112	0.981351
C20:2	0.0896149	0.962536
C15:0	0.0810028	0.891724
C17:1	0.0674316	0.981817
C24:1	0.0616207	-0.966817
C20:3(8.11.14)	0.0556506	0.993218
C20:1	0.0530076	-0.997918
C10:0	0.0505583	-0.99122
C22:1	0.0443531	-0.974631
C8:0	0.0220672	0.763873
C20:3(11.14.17)	0.0164719	0.253152
C4:0	0.00801769	-0.290296
C16:1	0.00586102	-0.248399

Table S6 Screening of fatty acid differentiators in flaxseed oil.

Compounds	VIP	S-Plot. p(corr)
C18:1	3.30511	0.992039
C18:3(9.12.15)	3.02616	0.951378
C18:0	2.29994	-0.975532
C18:2	1.69624	0.961175
C16:0	0.57923	0.829284
C20:1	0.349166	0.998602
C20:0	0.333787	0.997722
C22:6	0.271857	-0.990618
C22:0	0.149065	0.965594
C24:0	0.147562	0.992828
C17:0	0.0999989	0.957816
C8:0	0.0930847	-0.949879
C22:1	0.0920558	-0.99034
C20:2	0.0797407	0.919654
C22:2	0.0754512	-0.940512
C20:3(11.14.17)	0.0691228	0.995993
C24:1	0.0604218	0.995348
C15:0	0.0537098	0.907287
C20:4	0.0487141	-0.779598
C16:1	0.0483498	0.96205
C13:0	0.0469771	0.997226
C10:0	0.0465431	0.986254
C12:0	0.0383343	0.86945
C23:0	0.0365989	0.761806
C14:0	0.0314541	0.485234
C4:0	0.0248985	0.791345
C20:3(8.11.14)	0.0235613	0.914925
C11:0	0.015085	0.62365
C17:1	0.011151	0.541689

Table S7 Contribution rate of variables on loading plot.

Compounds	PC1	PC2
PC 16:0-18:3+AcO	0.043367	0.009877
Cer 18:1-14:0	0.043335	0.015628
DAG 14:0-20:0	0.043308	0.013481
PI 14:1-22:3	0.043307	0.012571
TAG 44:1-FA16:1	0.043302	0.01564
PE-P 16:0-18:2	0.043299	0.014199
TAG 47:0-FA14:0	0.043294	0.014103
TAG 58:7-FA22:6	0.043278	0.013757
Cer 18:1-18:0	0.043264	0.015504
TAG 56:4-FA22:4	0.043248	0.014135
FFA 26:1	0.043244	0.014013
BMP 18:1-22:2	0.043241	0.012532
BMP 18:2-22:2	0.043239	0.014015
HexCer 18:1-16:0	0.043225	0.009446
Hex2Cer 18:0-22:0	0.043215	0.017535
PI 18:1-22:2	0.043215	0.016665
TAG 58:8-FA20:3	0.043205	0.01044
DAG 18:2-22:4	0.043203	0.014192
HexCer 18:0-22:1	0.043197	0.014829
TAG 58:2-FA18:1	0.043197	0.01373
TAG 58:10-FA20:5	0.043193	0.015817
PS 16:1-18:4	0.043192	0.008592
PI 16:0-24:3	0.04319	0.015325
TAG 49:1-FA16:1	0.043172	0.012987
TAG 58:6-FA20:4	0.043172	0.019065
TAG 50:1-FA18:0	0.043169	0.015496
MGDG 16:1-24:1	0.043164	0.013512
TAG 54:8-FA20:5	0.043162	0.016868
PG 18:2-18:3	0.043158	0.013535
TAG 46:1-FA14:0	0.043149	0.014602
PI 16:0-22:1	0.043148	0.01556
TAG 50:3-FA16:1	0.043146	0.014008
PG 18:3-22:6	0.04313	0.011157
Hex2Cer 18:1-24:1	0.043124	0.015619
HexCer 18:1-20:0	0.043118	0.015377
DAG 18:2-20:5	0.04311	0.00788
CE 20:2	0.043108	0.012466
TAG 52:7-FA22:6	0.043079	0.018319
PS 18:1-22:1	0.043077	0.020303
PS 16:1-20:1	0.043075	0.01721
TAG 48:0-FA18:0	0.043074	0.017296
TAG 50:2-FA18:2	0.043067	0.016238

TAG 54:4-FA20:4	0.043059	0.018013
TAG 44:1-FA14:0	0.043053	0.013656
PS 18:3-20:1	0.043052	0.017948
TAG 50:1-FA18:1	0.04304	0.012405
TAG 56:7-FA18:0	0.043039	0.012876
TAG 56:7-FA18:2	0.043032	0.0217
TAG 58:7-FA18:0	0.043027	0.018484
PI 16:1-20:2	0.04302	0.02096
TAG 56:4-FA20:4	0.043009	0.019405
TAG 56:7-FA22:6	0.043009	0.020279
PG 18:2-20:4	0.043009	0.015657
TAG 50:1-FA20:1	0.043005	0.014745
TAG 52:5-FA20:5	0.043004	0.014001
PI 20:3-24:5	0.043001	0.014104
PI 18:2-24:0	0.043	0.019234
PC 14:0-20:2+AcO	0.042999	0.019337
DGDG 18:0-18:0	0.042982	0.017066
TAG 49:2-FA16:1	0.042981	0.015956
PI 18:0-24:1	0.04298	0.012799
TAG 49:1-FA17:0	0.042978	0.01684
TAG 48:0-FA16:0	0.042978	0.014083
BMP 18:2-20:4	0.042977	0.017398
TAG 58:8-FA20:4	0.042975	0.014652
FFA 24:4	0.04296	0.012516
TAG 51:0-FA16:0	0.042958	0.008557
TAG 53:6-FA20:4	0.042957	0.018388
PI 18:0-22:2	0.042954	0.019883
DGDG 14:1-20:0	0.042945	0.017507
BMP 18:1-20:1	0.042939	0.009831
TAG 42:0-FA16:0	0.042938	0.009341
TAG 58:8-FA22:6	0.042937	0.007296
Hex2Cer 18:0-18:1	0.042933	0.017966
TAG 56:8-FA22:5	0.042933	0.010129
TAG 48:2-FA18:2	0.042929	0.019303
Cer 18:0-24:1	0.042928	0.020222
TAG 52:6-FA16:1	0.042922	0.022459
PC 18:0-16:1+AcO	0.042913	0.020728
TAG 52:4-FA18:2	0.042911	0.023452
HexCer 18:0-20:0	0.042899	0.021358
PE-P 16:0-18:1	0.042897	0.020146
DGDG 16:1-18:2	0.042893	0.013837
Cer 18:1-16:1	0.042884	0.01798
TAG 47:2-FA14:0	0.042882	0.013652
LPC 16:0+AcO	0.04288	0.018518

TAG 54:4-FA22:4	0.042876	0.021014
FFA 26:6	0.042868	0.014392
Hex2Cer 18:0-26:0	0.042866	0.019618
TAG 48:1-FA16:0	0.042863	0.016401
TAG 58:8-FA18:1	0.042857	0.015296
DGDG 12:1-14:1	0.042854	0.019876
TAG 58:7-FA22:4	0.042852	0.018806
TAG 48:3-FA18:3	0.042836	0.017255
PE-P 16:1-18:1	0.042832	0.017182
TAG 48:1-FA18:0	0.042831	0.018776
Cer 18:0-24:0	0.042828	0.019006
TAG 48:4-FA20:4	0.042826	0.018195
BMP 16:0-22:5	0.042825	0.018318
PI 18:3-22:0	0.042818	0.016392
LPS 18:0	0.042816	0.015033
TAG 54:8-FA22:6	0.042812	0.019459
PS 18:1-22:3	0.042805	0.020472
Cer 18:0-20:0	0.042792	0.019392
BMP 16:1-20:1	0.04279	0.020768
PE-P 16:0-22:4	0.042788	0.011722
BMP 14:2-16:3	0.042785	0.020829
PI 22:0-22:0	0.04278	0.01977
DGDG 16:0-16:0	0.042779	0.019256
HexCer 18:1-26:0	0.04277	0.01919
TAG 50:3-FA18:2	0.042769	0.015011
PS 16:0-20:0	0.042766	0.01703
BMP 18:2-20:1	0.042756	0.014494
TAG 50:2-FA18:0	0.042753	0.012684
TAG 50:1-FA14:0	0.042712	0.020649
TAG 52:6-FA22:6	0.042712	0.018178
PI 18:1-24:3	0.042711	0.021368
CE 20:4	0.04271	0.022077
TAG 50:3-FA14:0	0.042702	0.021458
DGDG 16:0-16:1	0.042691	0.017008
PC 20:0-20:2+AcO	0.042687	0.022091
CE 18:2	0.042673	0.022181
TAG 52:4-FA20:3	0.042667	0.015432
Hex2Cer 18:1-26:0	0.042666	0.02426
PG 18:1-20:1	0.04266	0.0211
Hex2Cer 18:1-26:1	0.042651	0.022921
TAG 47:2-FA18:1	0.042638	0.020754
DAG 18:2-22:6	0.042626	0.020685
HexCer 18:1-26:1	0.042602	0.01775
TAG 60:10-FA22:6	0.042593	0.024457

Hex2Cer 18:0-24:0	0.042562	0.01959
TAG 44:3-FA18:2	0.042549	0.018046
TAG 50:4-FA20:4	0.042547	0.019052
CE 24:0	0.042536	0.016791
SM 16:0	0.042532	0.019472
TAG 50:2-FA18:1	0.042475	0.02068
TAG 56:7-FA22:5	0.04246	0.024117
TAG 44:2-FA14:0	0.042443	0.02038
TAG 50:4-FA18:3	0.042441	0.016191
PS 18:0-20:0	0.04241	0.027781
TAG 46:3-FA16:1	0.04228	0.020335
TAG 52:4-FA22:4	0.042266	0.025122
PS 20:1-20:4	0.04222	0.026635
HexCer 18:0-26:1	0.042148	0.028827
MGDG 18:2-24:1	0.042107	0.027203
TAG 42:2-FA18:2	0.041869	0.021374
TAG 50:3-FA18:3	0.041862	0.023109
TAG 44:0-FA14:0	0.041659	0.035371
TAG 46:0-FA14:0	0.041552	0.019749
Hex2Cer 18:0-20:0	0.041545	0.031798
PC 16:0-18:1+AcO	0.041432	0.023243
DGDG 18:0-18:5	0.041382	0.022419
TAG 49:3-FA18:2	0.041334	0.022324
DAG 18:2-20:4	0.041268	0.033408
TAG 42:1-FA14:0	0.041162	0.035793
TAG 46:2-FA16:1	0.04116	0.033224
TAG 40:0-FA16:0	0.041136	0.026191
PS 16:1-22:2	0.041061	0.03032
BMP 18:2-20:6	0.041015	0.021089
TAG 52:2-FA14:0	0.04098	0.018092
TAG 51:4-FA18:2	0.040943	0.019205
FFA 22:5	0.040923	0.027235
Cer 18:1-22:0	0.040627	0.033785
TAG 57:2-FA18:1	0.0406	0.026643
MGDG 18:2-24:0	0.040579	0.028954
Hex2Cer 18:1-22:0	0.04044	0.034176
DAG 18:2-20:3	0.040422	0.003477
TAG 46:4-FA18:2	0.040335	0.031589
PI 18:1-24:2	0.040211	0.032523
TAG 44:2-FA18:1	0.040102	0.029559
TAG 46:2-FA18:2	0.039977	0.022471
DAG 16:0-20:4	0.039976	0.033613
TAG 46:1-FA16:1	0.039788	0.022296
PG 16:0-20:5	0.039685	0.022852

PC 20:0-20:3+AcO	0.039664	0.004433
TAG 51:3-FA16:1	0.039632	0.029559
FFA 28:4	0.039422	0.04294
TAG 46:3-FA18:2	0.039357	0.050475
PS 18:1-18:2	0.039317	0.043832
FFA 26:5	0.039184	0.035773
TAG 44:2-FA18:2	0.038705	0.034585
TAG 42:1-FA16:0	0.038664	0.044595
TAG 52:3-FA14:0	0.038553	0.044845
TAG 42:1-FA18:1	0.038529	0.046756
FFA 20:5	0.038496	0.015565
TAG 44:2-FA16:1	0.038292	0.018214
TAG 54:8-FA20:4	0.038	0.057227
TAG 56:6-FA20:2	0.037882	0.039602
DGDG 12:0-16:0	0.037818	0.022038
BMP 16:0-20:5	0.03644	0.04791
TAG 54:4-FA16:1	0.036437	0.034741
TAG 47:1-FA16:1	0.036411	0.054859
TAG 52:1-FA20:0	0.036174	0.066629
TAG 52:1-FA20:1	0.036047	0.022262
PS 18:1-20:1	0.035495	0.029676
LPE 16:0	0.035413	0.054558
TAG 58:7-FA16:0	0.035211	0.011617
TAG 46:1-FA18:1	0.034777	0.063825
TAG 46:0-FA16:0	0.034589	0.053748
TAG 54:5-FA20:4	0.034215	0.044456
TAG 51:3-FA18:2	0.034058	0.036638
TAG 58:7-FA18:1	0.034034	0.057246
FFA 26:4	0.033847	0.066653
TAG 56:9-FA20:4	0.03381	-0.01226
HexCer 18:0-24:1	0.033512	0.054506
TAG 56:10-FA18:2	0.033056	0.070162
TAG 49:2-FA18:2	0.032663	-0.00517
PG 18:1-20:0	0.032193	0.063495
TAG 52:2-FA20:1	0.032122	0.061834
TAG 57:3-FA18:2	0.031948	0.044162
TAG 54:6-FA20:4	0.031244	0.080813
TAG 54:5-FA16:1	0.030908	0.049768
TAG 44:2-FA16:0	0.030907	0.046472
TAG 54:3-FA20:1	0.03081	0.038151
TAG 54:5-FA22:4	0.030575	0.031935
FFA 24:2	0.030535	0.06425
TAG 52:2-FA20:0	0.030518	0.061088
Cer 18:0-16:0	0.030385	0.067062

FFA 28:2	0.030251	0.065591
TAG 55:2-FA18:2	0.02976	0.052605
TAG 49:2-FA17:0	0.029427	0.037916
TAG 52:5-FA20:3	0.028881	0.088111
Cer 18:1-24:1	0.028449	0.074693
PE 18:2-18:2	0.028255	0.066343
PI 18:1-24:1	0.028185	0.015664
TAG 46:3-FA18:1	0.028123	0.017533
BMP 16:0-22:3	0.027924	0.070624
TAG 54:6-FA16:1	0.027831	0.074619
MGDG 18:1-22:1	0.027762	0.071222
SM 26:1	0.026395	0.055586
FFA 16:0	0.025201	0.038537
DGDG 14:0-18:0	0.025077	0.038398
TAG 56:3-FA18:1	0.02491	0.046179
TAG 49:2-FA16:0	0.024532	0.082156
Hex2Cer 18:1-20:0	0.024423	0.043376
TAG 49:0-FA16:0	0.02433	0.086688
TAG 56:3-FA20:2	0.023405	0.036002
FFA 30:3	0.022839	0.070795
FFA 24:1	0.022664	0.05504
TAG 54:5-FA16:0	0.021802	0.056489
MGDG 18:2-22:0	0.021572	0.100728
TAG 58:9-FA18:2	0.021554	0.064365
PS 16:0-22:1	0.017131	0.10109
TAG 51:2-FA18:2	0.016546	0.047991
BMP 16:0-16:3	0.015415	0.079224
TAG 56:4-FA18:2	0.01533	0.064747
TAG 53:4-FA18:2	0.015271	0.070593
TAG 55:1-FA16:0	0.013931	0.094651
DGDG 14:0-18:1	0.01283	0.09829
PG 16:0-16:3	0.012008	0.038239
TAG 48:4-FA18:2	0.011286	0.101765
PI 18:0-20:6	0.010343	0.072338
TAG 53:3-FA16:0	0.010142	0.07265
TAG 55:5-FA18:2	0.009859	0.076455
TAG 51:2-FA17:0	0.009785	0.075086
TAG 56:3-FA18:2	0.009438	0.040415
TAG 52:5-FA16:1	0.009283	0.058337
FFA 16:1	0.008988	0.000389
TAG 54:5-FA22:5	0.008178	0.083648
TAG 49:3-FA16:1	0.005508	0.052625
TAG 52:2-FA18:0	0.005433	0.106516
TAG 54:6-FA16:0	0.005432	0.082417

TAG 54:5-FA18:2	0.005245	0.053324
BMP 22:3-22:4	0.004904	0.08488
TAG 52:4-FA18:0	0.004451	0.026878
FFA 22:4	0.004328	0.020915
FFA 22:6	0.004065	0.095776
TAG 56:7-FA20:4	0.001706	0.09743
TAG 53:2-FA18:2	0.001321	0.105948
TAG 58:10-FA22:6	−0.0000260903	0.072007
FFA 18:0	-0.00149	0.071498
TAG 50:2-FA16:0	-0.00174	0.098729
TAG 54:2-FA20:0	-0.00176	0.089464
PC 16:0-18:0+AcO	-0.00203	0.014864
FFA 14:0	-0.00262	0.112111
TAG 55:4-FA18:2	-0.00278	0.050745
TAG 56:4-FA20:1	-0.00292	0.017517
TAG 53:3-FA17:0	-0.00354	0.047118
TAG 44:1-FA16:0	-0.00396	0.100876
DAG 18:1-20:4	-0.00432	0.089945
PI 16:0-24:4	-0.00504	0.121408
TAG 52:3-FA20:1	-0.00522	0.025214
PG 16:0-16:4	-0.0055	0.071712
PI 18:1-24:5	-0.00582	0.103329
PS 14:1-20:0	-0.0061	0.036554
TAG 56:6-FA18:2	-0.00615	0.062947
TAG 50:2-FA14:0	-0.00686	0.08468
TAG 52:3-FA18:2	-0.00772	0.091628
FFA 28:1	-0.00861	0.078187
MGDG 16:0-24:0	-0.00879	0.041146
TAG 51:2-FA16:0	-0.00932	0.067444
TAG 58:3-FA18:1	-0.0098	0.079339
PG 18:2-18:4	-0.00992	0.03854
DGDG 12:1-14:3	-0.01091	0.058258
TAG 52:3-FA18:1	-0.01096	0.063509
TAG 52:3-FA20:0	-0.01101	0.086031
TAG 46:2-FA18:1	-0.01207	-0.00555
FFA 30:1	-0.01338	0.088476
TAG 48:2-FA14:0	-0.01375	0.002289
FFA 18:2	-0.01442	0.042876
TAG 51:5-FA18:2	-0.01526	0.077291
PS 18:0-18:1	-0.01554	0.029274
TAG 54:2-FA16:0	-0.01581	-0.00973
FFA 20:4	-0.01588	0.040447
TAG 49:1-FA18:1	-0.01595	0.058461
TAG 54:4-FA20:3	-0.01644	0.073529

PI 18:1-22:0	-0.01648	-0.0043
FFA 16:2	-0.01687	0.115937
TAG 56:3-FA20:1	-0.01805	0.05145
DAG 16:0-18:2	-0.01818	0.012963
TAG 49:3-FA16:0	-0.01854	0.082184
TAG 56:5-FA20:1	-0.01878	0.046302
TAG 54:7-FA18:3	-0.01895	0.055153
TAG 58:6-FA18:0	-0.0192	0.103184
PI 20:0-22:3	-0.02	-0.01903
TAG 53:3-FA18:2	-0.02074	0.07845
DAG 14:0-18:2	-0.02094	-0.03831
TAG 56:4-FA20:3	-0.02112	-0.02071
TAG 49:2-FA14:0	-0.02124	0.024534
TAG 56:2-FA16:0	-0.02139	0.039734
TAG 54:2-FA20:1	-0.02167	0.037747
TAG 52:4-FA16:0	-0.02188	0.074303
TAG 54:7-FA18:2	-0.02191	0.022921
MGDG 18:1-22:2	-0.02197	0.047653
TAG 56:4-FA20:2	-0.02232	-0.01294
BMP 18:0-22:4	-0.02251	0.065035
TAG 44:1-FA18:1	-0.02272	0.013789
TAG 55:7-FA22:6	-0.02337	0.026872
TAG 50:1-FA16:0	-0.02356	0.039034
TAG 54:3-FA18:0	-0.02372	0.016462
TAG 56:7-FA16:0	-0.02377	0.06913
TAG 53:4-FA17:0	-0.02442	0.095268
TAG 58:7-FA22:5	-0.02447	0.019219
TAG 54:4-FA18:2	-0.02451	0.093689
SM 24:1	-0.02477	0.028455
TAG 58:9-FA18:1	-0.0248	0.077586
TAG 52:4-FA20:0	-0.02517	0.021751
TAG 56:2-FA20:0	-0.02538	0.010755
TAG 56:8-FA18:2	-0.02544	0.046487
TAG 58:8-FA18:2	-0.02555	0.046706
MGDG 18:1-20:2	-0.02565	0.044867
PI 18:0-18:5	-0.02573	0.065103
BMP 18:2-18:4	-0.02583	0.032475
TAG 54:4-FA18:0	-0.02643	-0.01834
TAG 56:6-FA18:1	-0.02656	0.058329
TAG 54:8-FA18:3	-0.02658	0.037558
TAG 54:2-FA18:2	-0.02681	0.070978
TAG 56:8-FA20:4	-0.02687	0.052079
CE 14:0	-0.02692	0.036166
TAG 52:2-FA18:1	-0.02746	0.091786

TAG 54:3-FA20:2	-0.0277	0.019417
TAG 52:2-FA18:2	-0.02785	0.02427
TAG 54:5-FA18:0	-0.02822	0.077161
PS 20:3-22:5	-0.02861	0.046706
TAG 48:4-FA16:0	-0.02864	0.073931
TAG 54:7-FA18:1	-0.02896	0.056298
FFA 22:0	-0.02903	0.030624
TAG 48:1-FA14:0	-0.02905	0.053789
FFA 22:3	-0.0292	0.053319
TAG 52:2-FA16:0	-0.02945	0.001402
FFA 30:0	-0.02953	0.008077
DGDG 12:1-14:4	-0.02961	0.04522
TAG 52:1-FA18:0	-0.02963	0.03105
TAG 54:3-FA18:2	-0.02982	-0.01083
TAG 50:0-FA16:0	-0.02985	0.05389
TAG 46:3-FA16:0	-0.03015	-0.05726
TAG 49:2-FA18:1	-0.03025	0.084451
TAG 54:3-FA16:1	-0.03038	0.080903
TAG 51:1-FA17:0	-0.03044	0.060462
FFA 18:3	-0.03056	0.077927
FFA 22:1	-0.0308	0.083435
TAG 54:2-FA18:0	-0.03083	-0.01593
PS 20:3-22:2	-0.03089	0.01845
DGDG 16:0-18:1	-0.03093	0.061267
PS 18:1-22:2	-0.03094	0.073282
TAG 52:4-FA16:1	-0.03109	0.037417
PS 16:1-22:3	-0.03115	0.075098
TAG 52:2-FA16:1	-0.03117	0.075737
TAG 54:5-FA18:3	-0.03118	0.022898
TAG 52:6-FA16:0	-0.0315	0.041811
TAG 52:0-FA20:0	-0.03154	0.012637
TAG 56:2-FA18:0	-0.03167	0.070683
TAG 48:1-FA18:1	-0.03173	0.056632
TAG 54:1-FA20:0	-0.03192	0.008395
FFA 28:3	-0.03206	0.065337
TAG 53:2-FA16:0	-0.03207	0.0771
TAG 56:7-FA18:3	-0.03207	0.06622
PS 18:0-18:2	-0.03225	0.021115
DAG 14:0-14:0	-0.03235	0.017136
Cer 18:0-14:0	-0.03258	0.028675
PI 18:2-22:1	-0.03268	-0.02026
TAG 53:4-FA16:0	-0.03274	0.046117
TAG 56:6-FA18:0	-0.03282	0.063374
TAG 54:4-FA20:2	-0.03285	0.076081

TAG 52:1-FA16:0	-0.03295	0.071885
TAG 54:3-FA18:1	-0.03324	0.066857
DAG 14:0-16:1	-0.03329	0.005169
BMP 18:2-20:5	-0.03334	0.060432
TAG 54:6-FA18:3	-0.03342	0.04909
DAG 18:1-18:2	-0.03343	0.037542
TAG 49:1-FA16:0	-0.03359	0.053801
TAG 56:6-FA22:6	-0.0338	0.053512
TAG 51:2-FA18:1	-0.0339	0.026837
DGDG 12:0-18:2	-0.03402	0.050343
TAG 51:4-FA16:1	-0.03435	0.037141
BMP 20:3-22:3	-0.03435	0.027411
TAG 54:1-FA16:0	-0.03438	0.053287
TAG 52:3-FA16:0	-0.03462	0.034533
FFA 26:0	-0.03474	-0.0218
TAG 56:5-FA22:4	-0.03477	0.021567
TAG 55:4-FA18:1	-0.03484	-0.01899
TAG 54:5-FA20:3	-0.03516	0.040075
Cer 18:1-16:0	-0.03523	0.026166
PS 18:0-22:3	-0.03539	0.060651
MGDG 16:0-16:0	-0.03546	0.012205
TAG 48:3-FA18:2	-0.03565	0.041492
TAG 53:2-FA17:0	-0.03565	0.040964
TAG 54:8-FA18:2	-0.03573	0.027083
TAG 54:0-FA16:0	-0.03585	0.00925
TAG 52:6-FA18:3	-0.03592	0.017196
TAG 52:8-FA16:1	-0.03592	0.041112
FFA 24:0	-0.03594	0.031932
TAG 58:10-FA18:2	-0.0361	-0.00833
DAG 16:1-18:2	-0.03614	0.058246
DAG 18:2-18:3	-0.03646	-0.00899
TAG 56:3-FA20:0	-0.03652	0.019214
TAG 54:6-FA18:2	-0.03657	0.044628
TAG 51:1-FA16:0	-0.03676	-0.0342
TAG 47:2-FA18:2	-0.03707	0.035043
DAG 18:0-18:2	-0.03725	0.040371
TAG 58:7-FA18:2	-0.03727	0.049864
TAG 54:2-FA20:2	-0.03731	0.045083
MGDG 18:1-20:0	-0.03734	0.007613
TAG 54:6-FA20:3	-0.03736	0.046383
DGDG 14:1-16:6	-0.03745	0.043316
TAG 54:2-FA18:1	-0.03746	0.039074
FFA 18:1	-0.03751	0.021929
TAG 53:1-FA16:0	-0.03752	0.024037

TAG 48:4-FA14:0	-0.03755	0.044943
TAG 56:7-FA20:3	-0.03756	0.039642
TAG 54:5-FA18:1	-0.03757	-0.01216
PS 22:4-22:5	-0.03757	0.022336
PS 16:0-18:1	-0.03763	0.026123
PG 18:1-18:4	-0.0377	0.035877
TAG 56:5-FA18:2	-0.03775	0.047559
DAG 18:1-18:1	-0.03781	0.036835
TAG 51:4-FA18:3	-0.03802	0.001367
TAG 52:7-FA16:0	-0.03804	0.015228
FFA 20:0	-0.03812	0.005366
TAG 52:6-FA18:1	-0.03819	0.015387
TAG 46:3-FA18:3	-0.03819	-0.00823
MGDG 18:2-22:1	-0.03838	0.013572
TAG 56:6-FA22:4	-0.03841	0.014564
DAG 16:0-18:3	-0.03841	0.052358
TAG 54:1-FA20:1	-0.03853	-0.0072
TAG 56:6-FA20:5	-0.03861	0.017562
DAG 16:1-18:0	-0.03874	0.00183
TAG 58:6-FA22:4	-0.0389	0.018862
FFA 30:2	-0.03892	0.029578
FFA 26:2	-0.03911	0.01131
TAG 52:4-FA18:3	-0.03918	0.041566
PS 16:0-18:2	-0.03918	0.013221
DGDG 12:1-18:0	-0.03919	0.0062
TAG 56:1-FA18:1	-0.03922	0.032431
DAG 18:1-22:6	-0.03923	0.010086
HexCer 18:1-22:1	-0.03926	-0.00558
TAG 54:6-FA18:1	-0.03929	0.014857
TAG 51:1-FA18:0	-0.03941	-0.00271
Cer 18:0-22:0	-0.03944	0.03959
TAG 54:1-FA18:1	-0.03947	0.02856
TAG 56:5-FA18:0	-0.03951	0.037535
PI 18:2-22:0	-0.03953	0.012103
TAG 52:5-FA22:5	-0.03955	0.00904
TAG 50:5-FA18:2	-0.03955	0.01148
PI 16:0-22:3	-0.03957	0.009114
DAG 16:0-16:1	-0.0396	0.036471
TAG 49:1-FA14:0	-0.03968	0.01191
DAG 14:0-18:1	-0.03968	-0.00524
TAG 54:4-FA18:1	-0.0397	0.017616
TAG 52:0-FA16:0	-0.03971	0.037421
PS 16:1-20:0	-0.03971	0.0453
TAG 51:2-FA16:1	-0.03973	0.018765

TAG 46:3-FA14:0	-0.0398	-0.01735
SM 22:1	-0.03988	-0.00549
TAG 50:5-FA18:3	-0.0399	0.003801
TAG 49:3-FA18:3	-0.03993	0.005409
FFA 22:2	-0.03997	0.017095
TAG 56:2-FA20:1	-0.03999	0.009163
PC 18:0-18:0+AcO	-0.04007	0.017211
DAG 18:1-20:2	-0.04013	0.009077
TAG 58:5-FA18:1	-0.04015	0.019249
TAG 47:1-FA18:1	-0.04015	0.013139
TAG 54:4-FA18:3	-0.04015	-0.01048
TAG 55:1-FA18:1	-0.04017	-0.00244
PG 18:1-18:6	-0.04019	0.008405
TAG 52:2-FA20:2	-0.04019	-0.01102
TAG 54:1-FA18:0	-0.0402	0.036777
PG 18:1-18:3	-0.04022	0.031342
TAG 48:2-FA18:1	-0.04026	0.005886
FFA 20:1	-0.04026	0.007225
PS 18:1-18:1	-0.04029	0.026241
TAG 53:2-FA18:1	-0.0403	0.015689
DAG 16:0-18:1	-0.04033	0.005319
TAG 52:8-FA18:2	-0.04033	0.019666
TAG 51:3-FA18:3	-0.04033	0.023724
DAG 16:1-20:2	-0.04034	0.028153
TAG 52:5-FA18:2	-0.04035	0.022609
FFA 20:3	-0.04037	0.01739
Cer 18:1-18:1	-0.04048	-0.00163
TAG 58:6-FA22:5	-0.0405	0.03124
TAG 56:8-FA18:3	-0.04053	0.036941
TAG 54:3-FA16:0	-0.04065	0.026551
BMP 18:1-18:4	-0.04069	-0.00051
TAG 53:0-FA16:0	-0.0407	-0.00651
FFA 28:6	-0.04076	-0.02125
TAG 51:3-FA17:0	-0.04077	0.006588
PE 16:0-18:2	-0.04079	0.027854
BMP 18:1-20:5	-0.0408	-0.03286
TAG 55:3-FA18:1	-0.0408	0.0177
TAG 52:4-FA18:1	-0.04083	0.013996
MGDG 18:1-20:1	-0.04087	-0.00458
TAG 56:8-FA22:6	-0.04087	-0.01533
TAG 55:5-FA18:1	-0.04089	0.031377
DAG 16:1-16:1	-0.04091	0.038866
TAG 56:5-FA18:1	-0.04092	-0.00258
TAG 53:1-FA17:0	-0.04093	0.035125

PC 18:0-18:1+AcO	-0.04094	0.024171
TAG 52:7-FA18:1	-0.04098	0.011014
TAG 56:1-FA16:0	-0.04099	0.002664
PS 16:0-18:0	-0.04101	0.001232
DGDG 14:0-16:1	-0.04106	-0.01163
TAG 54:4-FA20:1	-0.04106	0.003431
TAG 42:1-FA16:1	-0.0411	0.032599
TAG 56:5-FA20:2	-0.04113	0.033171
TAG 56:8-FA16:0	-0.04116	-0.00997
PI 18:0-22:0	-0.04117	0.002908
MGDG 16:0-22:3	-0.04117	0.03762
DAG 18:0-18:1	-0.04118	0.015305
PG 18:1-20:5	-0.0412	-0.00396
MGDG 18:1-18:2	-0.04122	0.010782
PS 18:3-22:2	-0.04122	0.026397
FFA 20:2	-0.04123	0.0284
TAG 53:4-FA18:3	-0.04124	-0.00774
DAG 18:1-20:1	-0.04126	-0.02112
TAG 51:0-FA18:0	-0.04134	0.035808
TAG 56:5-FA22:5	-0.04134	0.008449
TAG 56:6-FA22:5	-0.04137	-0.01142
TAG 52:5-FA18:3	-0.04139	-0.01971
TAG 52:1-FA18:1	-0.0414	0.019554
TAG 44:0-FA18:0	-0.0414	0.01458
TAG 48:3-FA18:1	-0.04143	0.005922
TAG 54:3-FA18:3	-0.04144	-0.00572
MGDG 18:2-20:1	-0.04144	0.025556
TAG 52:5-FA18:1	-0.04148	-0.0184
TAG 53:1-FA18:0	-0.04149	0.009665
DAG 16:1-18:1	-0.04149	0.022917
TAG 54:3-FA20:3	-0.04151	0.01213
TAG 54:4-FA16:0	-0.04152	0.014665
FFA 24:3	-0.04156	0.008831
DAG 18:1-20:5	-0.04156	-0.00592
DAG 16:0-20:3	-0.04162	-0.02018
DGDG 14:0-14:0	-0.04163	0.019894
MGDG 16:0-24:1	-0.04164	0.015494
DGDG 12:0-18:1	-0.0417	0.01075
MGDG 18:0-18:3	-0.04172	0.033823
TAG 48:4-FA18:3	-0.04173	-0.01239
DAG 18:0-18:3	-0.04173	-0.02131
TAG 51:1-FA18:1	-0.04176	0.014718
TAG 56:5-FA20:4	-0.04182	0.016465
FFA 30:4	-0.04186	-0.00326

TAG 50:4-FA16:0	-0.04186	0.006953
TAG 50:5-FA14:0	-0.04187	-0.0097
TAG 52:7-FA20:5	-0.04194	0.020866
TAG 56:3-FA16:0	-0.04196	-0.01787
TAG 50:5-FA18:1	-0.04197	0.024521
DGDG 20:6-20:6	-0.04199	0.025
PG 16:1-20:1	-0.04201	0.010385
PS 22:1-22:2	-0.04202	0.004216
TAG 48:4-FA18:1	-0.04202	0.033497
PG 18:0-20:2	-0.04203	0.02291
PI 18:3-20:3	-0.04205	0.017157
BMP 18:1-18:3	-0.04205	0.007148
TAG 56:6-FA20:3	-0.04205	-0.00762
Cer 18:1-22:1	-0.04209	0.022262
FFA 24:6	-0.04209	0.00186
TAG 56:7-FA18:1	-0.04211	0.004219
TAG 52:5-FA16:0	-0.04212	0.015708
MGDG 16:0-22:0	-0.04212	0.014739
BMP 18:0-22:3	-0.04213	0.007275
FFA 30:6	-0.04213	-0.02202
DGDG 14:1-16:0	-0.04214	-0.0044
PC 16:0-16:0+AcO	-0.04214	-0.00153
DGDG 12:1-14:5	-0.04215	0.015207
TAG 52:6-FA14:0	-0.04217	0.026971
DGDG 16:1-18:0	-0.04218	0.001012
TAG 51:5-FA18:3	-0.04219	0.006984
Cer 18:1-14:1	-0.04221	-0.0264
PE 18:1-18:3	-0.04221	-0.00544
CE 16:1	-0.04222	-0.01169
TAG 52:3-FA18:3	-0.04222	0.023141
PS 18:1-20:0	-0.04223	0.01162
MGDG 16:0-24:2	-0.04223	0.028542
BMP 18:3-20:5	-0.04225	0.011906
BMP 18:2-18:2	-0.04226	-0.01193
TAG 54:5-FA20:2	-0.04229	-0.01278
CE 16:0	-0.04229	-0.006
CE 20:1	-0.0423	0.020541
PE-P 16:0-18:3	-0.04231	0.003403
PI 18:3-24:5	-0.04233	0.012745
FFA 26:3	-0.04234	0.019888
PG 16:0-22:2	-0.04237	-0.00562
TAG 50:4-FA18:2	-0.04237	0.008534
DAG 16:1-20:0	-0.04238	0.008695
PI 18:1-20:2	-0.04238	0.00524

TAG 50:5-FA20:5	-0.04238	0.019257
Hex2Cer 18:0-18:0	-0.04239	-0.00927
TAG 52:3-FA18:0	-0.04242	0.026359
MGDG 16:1-22:0	-0.04242	0.002185
BMP 18:1-18:2	-0.04243	-0.01092
DAG 16:1-18:3	-0.04245	-0.00017
TAG 50:2-FA16:1	-0.04245	-0.00322
TAG 56:4-FA16:0	-0.04247	0.017851
PI 18:0-20:2	-0.04248	0.014824
PS 22:2-22:3	-0.04248	0.013526
Hex2Cer 18:1-18:0	-0.04248	-0.01733
FFA 28:0	-0.04248	0.018501
MGDG 18:1-24:1	-0.04249	0.014545
CE 22:4	-0.04249	0.006842
PI 18:1-22:1	-0.0425	-0.0208
PS 18:3-22:1	-0.04251	-0.00272
TAG 56:4-FA18:1	-0.04251	0.017875
TAG 56:5-FA16:0	-0.04252	0.000881
TAG 49:0-FA18:0	-0.04253	-0.01365
TAG 52:6-FA18:2	-0.04255	0.011823
TAG 48:5-FA18:3	-0.04255	0.018553
TAG 48:4-FA16:1	-0.04255	0.011005
MGDG 16:0-22:2	-0.04255	-0.00027
TAG 48:3-FA14:0	-0.04255	-0.00585
PI 18:3-20:2	-0.04257	0.013981
PI 16:0-22:2	-0.04259	-0.01456
DGDG 16:1-20:1	-0.04259	-0.00071
DGDG 14:0-18:3	-0.04259	-0.00187
PS 16:1-22:5	-0.0426	0.003888
DGDG 12:0-20:0	-0.0426	0.021084
HexCer 18:0-22:0	-0.04264	-0.01454
BMP 20:1-22:4	-0.04265	-0.00787
Hex2Cer 18:1-20:1	-0.04266	0.012881
DAG 16:1-22:6	-0.04267	0.014475
TAG 58:9-FA22:6	-0.04269	0.018345
MGDG 18:1-24:0	-0.04269	0.007343
CE 22:2	-0.04269	-0.01207
TAG 52:6-FA20:5	-0.04269	0.002361
MGDG 18:1-22:0	-0.0427	0.010944
DGDG 16:0-20:2	-0.0427	0.003756
HexCer 18:1-20:1	-0.0427	-0.00576
LPE 18:0	-0.04272	-0.00861
PC 18:1-22:4+AcO	-0.04272	0.012566
TAG 60:12-FA22:6	-0.04273	-0.00285

PS 18:0-22:5	-0.04273	-0.00845
BMP 18:0-20:2	-0.04273	0.009985
PI 18:1-24:6	-0.04274	-0.00729
PI 18:0-24:4	-0.04276	0.005493
MGDG 18:0-20:1	-0.04279	-0.00697
PI 16:0-22:4	-0.04279	0.011601
PS 18:2-22:2	-0.0428	-0.009
CE 18:3	-0.04281	0.009136
TAG 48:3-FA16:0	-0.04282	0.001239
HexCer 18:1-14:0	-0.04282	0.014514
TAG 56:5-FA20:3	-0.04284	-0.00406
TAG 56:4-FA18:0	-0.04284	0.014148
PS 18:1-22:0	-0.04286	0.007019
PC 20:0-22:6+AcO	-0.04286	0.008581
TAG 48:5-FA18:2	-0.04289	0.005262
TAG 50:5-FA16:1	-0.0429	0.011426
TAG 56:6-FA18:3	-0.04291	0.000102
SM 26:0	-0.04291	0.01214
MGDG 18:2-20:0	-0.04291	-0.00047
TAG 50:3-FA16:0	-0.04291	0.011047
TAG 50:4-FA16:1	-0.04292	0.001773
PI 18:0-24:2	-0.04293	-0.0045
PE 16:0-18:1	-0.04296	-0.00855
PS 18:1-22:5	-0.04297	-0.01091
TAG 56:7-FA22:4	-0.04298	-0.01072
TAG 58:8-FA22:5	-0.043	0.002313
TAG 52:4-FA20:2	-0.04302	0.015458
DGDG 16:0-18:0	-0.04304	0.008364
DAG 16:0-20:5	-0.04304	-0.00237
PS 14:1-22:1	-0.04304	0.007678
FFA 28:5	-0.04307	0.008416
Cer 18:0-18:0	-0.04308	0.002444
DGDG 18:1-18:2	-0.04309	-0.01088
TAG 58:6-FA18:1	-0.0431	0.012218
PC 20:0-18:1+AcO	-0.04312	0.009845
MGDG 18:0-20:0	-0.04317	0.003466
CE 22:1	-0.04317	0.006368
HexCer 18:0-26:0	-0.04317	-0.00615
PS 18:0-20:3	-0.0432	0.004936
DAG 14:0-18:3	-0.0432	-0.00639
DAG 18:1-22:4	-0.04321	0.001613
PI 18:1-20:1	-0.04321	0.010673
TAG 50:5-FA16:0	-0.04323	-0.0032
PC 16:0-20:3+AcO	-0.04325	0.006899

PS 20:3-22:3	-0.04325	0.002636
DGDG 12:1-14:2	-0.04325	0.005842
PE-P 18:0-18:3	-0.04325	-0.00454
DGDG 16:0-20:3	-0.04327	0.007301
TAG 54:7-FA20:4	-0.04329	0.000538
TAG 52:3-FA16:1	-0.04329	-0.00634
TAG 52:0-FA18:0	-0.0433	-0.01199
TAG 51:0-FA17:0	-0.04332	0.012857
PS 18:3-22:3	-0.04335	0.005059
TAG 56:3-FA18:0	-0.04336	-0.00509
TAG 58:10-FA22:5	-0.04337	-0.00681
TAG 50:3-FA18:0	-0.04338	0.001678
PC 20:0-18:3+AcO	-0.04338	0.001286
PS 18:2-22:1	-0.04338	0.012824
Hex2Cer 18:1-22:1	-0.0434	-0.00108
Cer 18:0-26:1	-0.0434	0.004047
TAG 49:0-FA17:0	-0.04349	0.006535
DAG 18:2-22:5	-0.04352	0.004157
